# Molecular Human Targets of Bioactive Alkaloid-Type Compounds from *Tabernaemontana cymose* Jacq.

**DOI:** 10.3390/molecules26123765

**Published:** 2021-06-21

**Authors:** Andrés Oliveros-Díaz, Jesús Olivero-Verbel, Yina Pájaro-González, Fredyc Díaz-Castillo

**Affiliations:** 1 Laboratory of Phytochemical and Pharmacological Research, Zaragocilla Campus, School of Pharmaceutical Sciences, University of Cartagena, Cartagena 130014, Bolívar, Colombia; aoliverosd@unicartagena.edu.co; 2 Environmental and Computational Chemistry Group, Zaragocilla Campus, School of Pharmaceutical Sciences, University of Cartagena, Cartagena 130014, Bolivar, Colombia; joliverov@unicartagena.edu.co; 3 Research Group in Healthcare Pharmacy and Pharmacology, Faculty of Chemistry and Pharmacy, University of Atlántico, Barranquilla 080003, Atlántico, Colombia; yinapajaro@mail.uniatlantico.edu.co

**Keywords:** indole alkaloid, drug discovery, AutoDock Vina, STRING, signaling pathway

## Abstract

Alkaloids are a group of secondary metabolites that have been widely studied for the discovery of new drugs due to their properties on the central nervous system and their anti-inflammatory, antioxidant and anti-cancer activities. Molecular docking was performed for 10 indole alkaloids identified in the ethanol extract of *Tabernaemontana cymosa* Jacq. with 951 human targets involved in different diseases. The results were analyzed through the KEGG and STRING databases, finding the most relevant physiological associations for alkaloids. The molecule 5-oxocoronaridine proved to be the most active molecule against human proteins (binding energy affinity average = −9.2 kcal/mol) and the analysis of the interactions between the affected proteins pointed to the PI3K/ Akt/mTOR signaling pathway as the main target. The above indicates that indole alkaloids from *T. cymosa* constitute a promising source for the search and development of new treatments against different types of cancer.

## 1. Introduction

Plants are fundamental for all life on the planet because they provide oxygen, nutrients, and food, and play a key role in climate change processes by absorbing carbon dioxide (CO_2_) from the atmosphere [1,2]. Nonetheless, one of the most remarkable characteristics of plants is their ability to produce a wide diversity of molecules through their metabolism. These molecules, also known as secondary metabolites, are divided into several groups such as alkaloids, tannins, flavonoids, quinones, coumarins, lignans, terpenes and sterols, that participate in plant adaptation to the surrounding environment, reproduction and as a defense mechanism against pests and herbivores [3,4]. Secondary metabolites presence in plants gives them a broad range of biological activities, as a consequence, they have been used to treat diseases and eliminate pests for centuries, making them an important source of new molecules with pharmacological activity [5,6].

Colombia is the most biodiverse country in the world in proportion to its territorial area; a decade ago, it was considered that in the country there were around 50,000 plant species, however, recent studies have shown that the real range figures between 29,000 and 31,000 species [7]. Despite the richness of Colombian flora, a small number of species has been studied in order to discover their pharmacological characteristics and chemical composition [8,9,10,11]. Studies of natural products from plants have led to the discovery of widely used drugs, and it is considered that 52% of new molecules approved since 1981 for disease treatment are natural products or derived from these molecules [12]. Well known examples are morphine and codeine isolated from *Papaver somniferum* L. extract or some antidiabetic medications such as metformin, which is a derivative of galegine obtained from *Galega officinalis* L. [13].

The most famous compound derived from plants is a diterpenoid called paclitaxel; it was first isolated in 1963 from *Taxus brevifolia* Nutt. plant and used as a main anticancer agent until now [14]. Nevertheless, the discovery of natural products with the potential to become new drugs is a long and expensive process that consists of many stages [15,16]. Generally, they start with ethnobotanical studies, surveys and identification of medicinal plants, and then bio-directed phytochemical studies are conducted to isolate the bioactive compounds [17].

This year, the world has witnessed the longest pandemic in recent decades. The new coronavirus (SARS-CoV-2) was first detected in Wuhan, China, and it is spreading across the planet at unprecedented speed, causing more than 2.9 million deaths so far [18,19]. Unfortunately, the severity of this pandemic only reveals the lack of knowledge regarding new medications to counteract the impacts of emerging diseases and others already known as multi-resistant bacteria infections, leishmaniasis, tuberculosis, Human Immunodeficiency Virus (HIV), dengue, Zika, among others [20,21,22,23,24].

Drug discovery is a time-consuming and expensive process; as a solution, the application of computational approaches and bioinformatics tools has boomed due to the volume of information that can be handled and the proper results obtained regarding to therapeutic targets for the molecules evaluated [25,26]. Furthermore, in silico studies can provide data about structure-activity relationships, possible binding sites, toxicity and pharmacology networks without incurring high research costs [27,28,29]. Through molecular docking the complementarity between a molecule and its target protein could be determined; this approach in conjunction with other bioinformatic tools allows to identify protein–protein interaction networks (PPIs) and, in this context, it is possible to evaluate the ability of new molecules to disturb or cause abnormalities on biological pathways in order to analyze their pharmacological activities [30,31].

Natural products have proven to be one of the main sources of new drugs and, the current situation proposes a favorable scenario for the search for new molecules with potential biological activity for diseases treatment. In our investigations, we have studied the chemical composition of 39 plant species from the Colombian Caribbean region [32], throughout these investigations, the specie *Tabernaemontana cymosa* Jacq. proved to be an indole alkaloid-rich plant. Additionally, these plant have shown promising effects against vectors of viral diseases such as *Aedes aegypti* L. mosquito, as well as antiviral and anti-proliferative activity against dengue virus and lung cancer cell lines respectively [8,33,34]. The present work aims to discover the molecular human targets of bioactive alkaloids present in *Tabernaemontana cymosa* extract and its potential medicinal use. For this reason, in the current study we evaluated the binding potential of 10 indole alkaloids found in *T. cymosa* with human proteins involved in different diseases processes, using molecular docking approach and analysis of PPIs through databases such as STRING and KEGG Pathway.

## 2. Results

### 2.1. Plant Material and Compound Identification 

**Figure 1:**
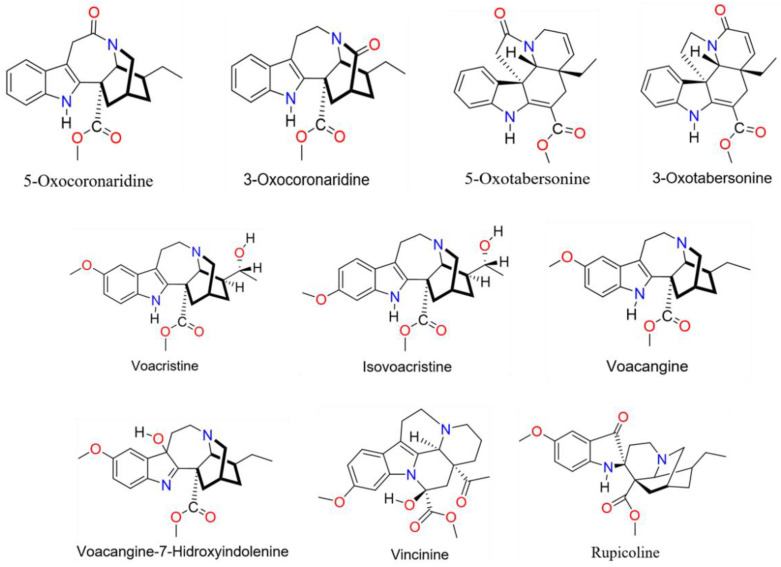
Molecular structure of indole alkaloids presents in *Tabernaemontana cymosa* Jacq.

A reddish-brown color extract was obtained (75 g) with a yield of 10.85%, a total of 23 molecules of the indole alkaloid type were isolated and identified from the ethanolic extract of *Tabernaemontana cymosa*. Alkaloids were detected in the peaks comprised in the retention times between 9 and 21 min, the molecules used for virtual screening are shown in Figure 1. The LTQ Orbitrap database (ThermoElectron-Corporation) suggested the 23 chemical structures for the alkaloids identified previously (Table 1), based on their mass/charge ratio (*m*/*z*).

### 2.2. Virtual Screenig for Identified Alkaloids

**Figure 2:**
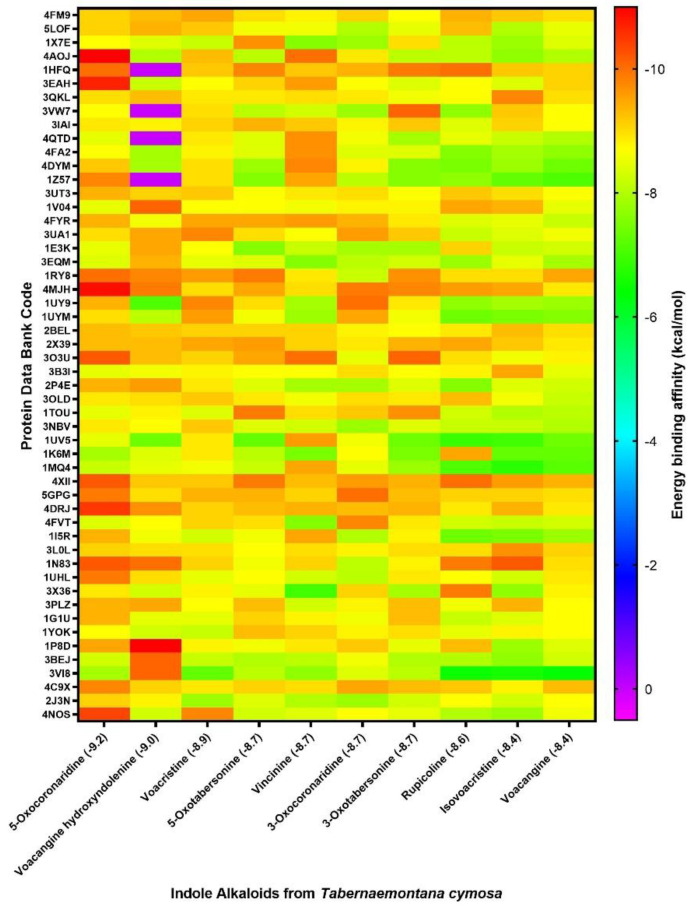
Heat-map of the molecular docking affinities values (kcal/mol) for indole alkaloids with human proteins. Values in parenthesis indicate the average binding energy for each molecule.

The 10 selected alkaloids showed good affinity scores with human proteins, as shown in Table 2, molecules such as voacangine-7-hydroxydolenine, voactristine and 3-oxotabersonine form highly stable complexes with important human proteins such as nitric oxide synthases (NOS) and dihydrofolate reductase (DHFR). The heat map shown in Figure 2 gives a broader picture of the behavior of the 10 molecules regarding to human targets. In the butyrylcholinesterase (4XII; BCHE) row, a heat bar (red-orange color) can be observed, showing that all the molecules have a high affinity with this protein. The main function of BChE is to protect the central nervous system (CNS) by contributing to the inactivation processes of the molecules that inhibit neurotransmitter acetylcholine [48]. Therefore, its inhibition can modulate the activity of the nervous system by affecting the transmission of neuronal signals. These heat bars can also be observed in the rows of proteins associated with the mTOR signaling pathway such as FK506 (5GPG) and the mTOR kinase (4DRJ) itself, directly affecting metabolism, protein translation, cell growth and proliferation [49]. The hot zones on the map indicate that 5-oxocoronaridine and voacangine-7-hydroxyndolenine obtained the best affinity scores for most of the evaluated targets, on the contrary, rupicoline, voacangine and isovoacangine molecules presented green zones that indicate a lack of affinity with human targets.

5-Oxocoronaridine compound obtained the best binding energy values for human proteins (Table 2) obtaining 32 ligand-protein complexes with strong affinity energies (<−9.0 kcal/mol). Within the 5-oxocoronaridine targets, we were able to find four oxido-reductase class proteins related to the oxidative metabolism of reactive nitrogen species such as oxide nitric synthases 2 and 3 (NOS2, NOS3) and dihydrofolate reductase (DHFR), in addition to catalytic enzymes such as aldo-keto reductase (AKR1C3). However, the most affected protein by this molecule was the High affinity nerve growth factor receptor or NTRK1 (−11.0 kcal/mol), which also obtained the highest affinity score recorded for all molecules. The results of the molecular docking of the 10 indole alkaloids against the 951 human proteins are shown in the Supplementary Material (Table S1). Voacangine-7-hydroxyndolenine and voacristine were the following most active molecules, with 26 and 27 protein complexes that exceeded −9.0 kcal/mol. The oxysterols LXR-beta receptor (1P8D) is the most significant target voacangine-7-hydroxyndolenine with a negative binding energy of −11.0 kcal/mol, indicating a potent inhibition of this protein and possibly affecting inflammation and tumorigenesis processes in the organism [50].

### 2.3. Protein–Protein Interactions (PPIs)

**Figure 3:**
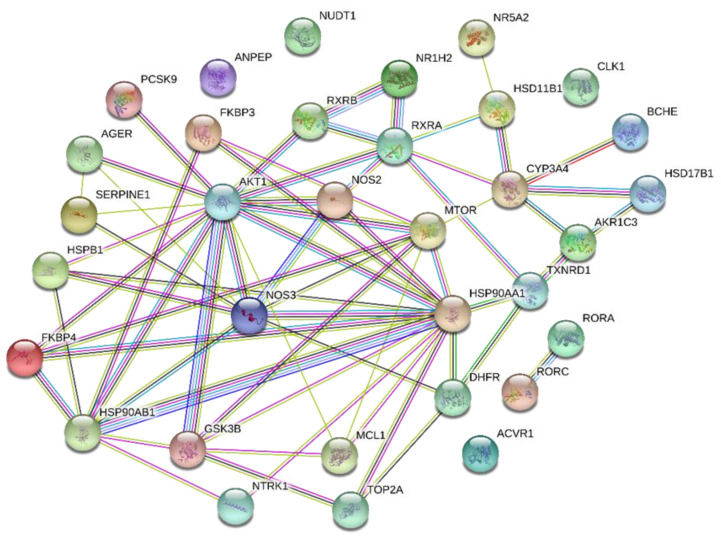
Predicted protein interaction network for complexes with 5-oxocoronaridine alkaloid (PPI enrichment *p*-value: 3.45 × 10^−12^). Edges represent the evidence of protein–protein associations. Nodes represent proteins with good binding affinity energies (lower than −9.0 kcal/mol) with the molecule. The names of the proteins represented in each node and their respective acronyms can be found in Supplementary Material Table S3.

In order to analyze the most relevant protein–protein interactions for indole alkaloids, we chose the molecule with the best binding behavior against the human targets tested. 32 hits were obtained from protein complexes with 5-oxocoronaridine according to the established parameters (affinity binding energy between −9.0 and −11.0 kcal/mol). According to the results, AKT1 was determined as the main core of the network and two secondary cores belonging to the MTOR and HSP90AA1 proteins (See Figure 3). The aforementioned major nodes have predicted functional interactions with many of the proteins inhibited by 5-oxocoronaridine alkaloid, among them, connections with NOS3 and NOS2 predominate. The most stable complex for this molecule was calculated with the NTRK1 protein (−11.0 kcal/mol), which is outside of the greatest interaction area of the network. However, important protein interactions are seen in the AKT1 signaling cascade, which has been the most affected by the activity of the alkaloid. PPIs for voacristine and voacangine-7-hydroxyndolenine complexes are also discuss due to their important theorical activity, this information is shown in Supplementary Material Figure S1 and Figure S2, respectively. AKT1 and NOS3 are also important nodes in both molecules PPIs.

### 2.4. Genomic Association

**Figure 4:**
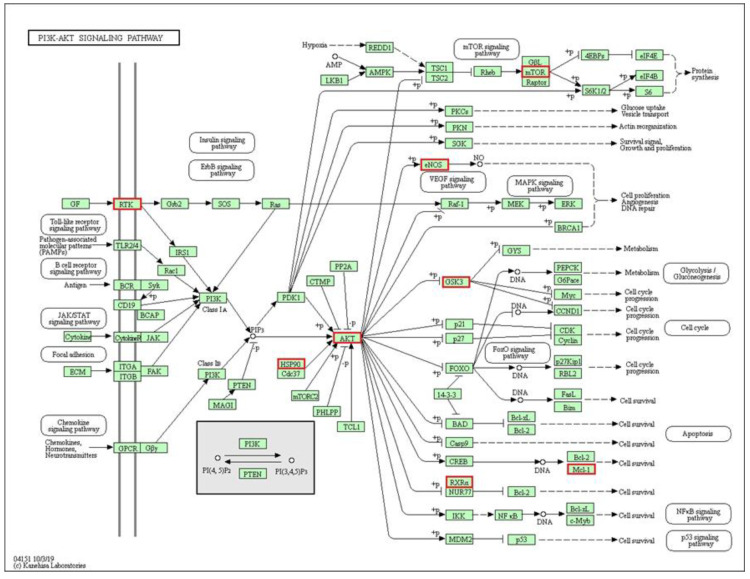
KEGG pathway for proteins associated to 5-oxocoronaridine in PI3K-AKT signaling pathway. Red squares represent potential targets of the molecule.

To identify how the indole alkaloid 5-oxocoronaridine affects the biological processes related to the interactions of the protein network obtained through the STRING database, we used the analysis of functional enrichments in the network in order to find the affected signaling cascades. In this way, the STRING result connects to the KEGG Pathway database, where we found that the proteins in the PPIs network are related to the PI3K-AKT signaling pathway (false rate discovery = 3.69 × 10^−7^). In this study, we found that 5-oxocoronaridine inhibits the function of eight proteins that actively participate in this signaling cascade (indicated by red boxes in Figure 4).

The eight inhibited proteins by 5-oxocoronaridine are RTK, AKT, HSP90, GSK3, RXRA, Mcl-1, eNOS and mTOR, which perform functions at different levels of the cascade and as a consequence the effects of this indole alkaloid can be seen reflected in all the processes regulated by this signaling pathway such as apoptosis, DNA repair, cell cycle, gluconeogenesis and protein synthesis. The disruption is even more significant due to the inhibition on AKT1 (−9.0 kcal/mol), being the main controller of the signaling cascade due to its phosphorylation and inhibition effects upstream.

## 3. Discussion

In silico studies performed for the 10 indole alkaloids revealed that these secondary metabolites family has a binding potential to important human target proteins. The theoretical affinity values of the molecules against the 951 proteins show a broad spectrum of activity, since they have the ability to interact with targets in various physiological processes (Supplementary Material, Table S1). The affinity values ranged from very stable interactions (−11.0 kcal/mol) to null associations (lower than −2.0 kcal/mol).

Although 5-oxocoronaridine, voacangine-7-hydroxyndolenine and voacristine are the three molecules that possess the best global affinity energies with respect to the evaluated targets, the other alkaloids also showed significant global averages (lower than −8.0 kcal/mol). The best complexes of each molecule were displayed as a heat map (Figure 2), showing significant differences in the targets of each alkaloid. The heat zones are scattered throughout the graph, however some of them can be viewed in horizontal section indicating the affinity of all alkaloids tested for a particular target. Alkaloids are natural products known for their neurological activity, which inhibit essential enzymes for the transmission of nerve signals such as acetylcholinesterase (AChE) and butyrynylcholinesterase (BChE) [51,52]. This coincides with our findings because one of the most notable heat zones is that corresponding to proteins related to neuroprotection processes (5GPG; 4DRJ) including BChE (4XII) and Nerve growth factor receptor (4AOJ).

The studied molecules are characterized by their bicyclic structure consisting of a benzene ring fused to a pyrrole ring with a nitrogen, however, the third ring and the position of the common functional groups in them confer very different biological activities [53]. An example of this situation is the disparity of the affinity scores between voacristine and isovoacristine, whose only structural difference is the position of the methoxyl group on the benzene ring. This change causes voacristine to be the third molecule with the best global affinity average (−8.9 kcal/mol), while isovoacristine is one of the molecules with the poorest scores. The protein binding behavior in the heat map denotes a low activity for isovoacristine. This may mean a change in the intermolecular interactions of the indole group with the amino acids of the binding domain of human proteins.

Functional interactions and effects on signaling pathways analysis were carried out using the 5-oxocoronaridine molecule, which gave the higher affinity values. Among the main targets of this alkaloid, we found interactions between proteins such as AKT1 (3QKL), NOS3 (3EAH), NOS2 (4NOS), HSP90 (1UY9) and nuclear receptors such as RXRA (1G1U). Furthermore, the highest affinity score was obtained for the complex 5-oxocoronaridine/NTRK1 (−11.0 kcal/mol), which is essential for the function of the nervous system mediating processes such as nervous cell development, growth and differentiation.

All these proteins are involved in the PI3K-Akt signaling pathway; therefore, their inhibition or activation could cause disruption in processes such as apoptosis, cell differentiation and proliferation, angiogenesis, protein synthesis, DNA repair and metabolism [54,55]. Furthermore, the binding effect of 5-oxocoronaridine on AKT1 could have important biological activity by inhibiting the molecular function of apoptotic and coagulation proteins such as MCL1 and SERPINE1 respectively, which could lead to uncontrolled cellular proliferation and cardiac issues [56].

ATK1 also mediates the activation of NOS3 producing an increase in Nitric Oxide increasing the possibilities of oxidative stress and regulating protein synthesis through the phosphorylation of protein complexes in the mTOR signaling cascade [57,58]. On the other hand, the protein–protein interactions of HSP90 are due to the binding and catalysis of their alpha and beta units that play an important role in the regulation of AKT1 and the preservation of the functional conformation of other proteins as a cell response to stress [59,60].

The PI3K-Akt signaling pathway is one of the best characterized pathways, its regulation has been widely studied due to its influence on neurodegenerative and carcinogenic processes [61,62]. Research by Shrivastava et al. has shown the in silico and in vitro anticancer activity of alkaloids capable of inhibiting the mTOR and PI3K proteins, thus inactivating the Akt1 signaling pathway [63]. Our data show an even greater inhibition of mTOR than that obtained by Shrivastava et al. (−7.1 kcal/mol) reaching an affinity value of −10.5 kcal/mol. Consequently, indole alkaloids such as 5-oxocoronaridine emerge as promising molecules for anticancer studies. The use of computational tools and databases such as KEGG allowed us to identify other 5-oxocoronaridine potential targets within PI3K-Akt signaling cascade (Figure 4) for the selected affinity cut-off (<−9.0 kcal/mol).

Interestingly, the inhibitory action of this molecule occurs at various levels of the signaling cascade, one of its most important complexes is obtained with the NRTK1 transmembrane receptor (4AOJ; −11.0 kcal/mol). It also has strong interactions with cofactors such HSP90 (1UY9; −9.4 kcal/mol) and AKT, the enzyme responsible for critical phosphorylation processes in this pathway (3QKL; −9.0 kcal/mol). Some Stemona alkaloids are capable of preventing the progression of serious lung diseases by regulating the levels of AKT1 and PI3K through direct interaction with these proteins [64].

On the other hand, the antiproliferative activity of the pyrrolopyrimidine-type alkaloids has been studied based on their inhibitory action on AKT1 (IC_50_, 1.5 ± 1.3 Nm), as well as other types of alkaloids such as those of quinolizidine and isoquinoline that provide anticancer activity through regulation of PI3K/Akt/mTOR signaling [65,66]. As a consequence of the high affinity of 5-oxocoronaridine alkaloid with enzymes such as AKT and mTOR kinase (4DRJ; −10.5 kcal/mol) and its influence on apoptotic processes and cell survival, we think that indole alkaloids of the iboga type are promising molecules for future anticancer studies. In the same way, the PPIs network that we performed showed the interaction capacity of all the molecules tested with important proteins involved in the functioning and maintenance of the CNS, which makes them interesting substances for study as a possible treatment of neurodegenerative diseases.

A robust review of the biological properties of isolated indole alkaloids of the genus *Tabernaemontana* has recently been reported, where up to six of the compounds used in the present study are mentioned as anticancer molecules (3-oxocoronaridine, 5-oxocoronaridine, voacristine, isovoacristine, voacangine and voacangine-7-hydroxyndolenine); in addition to the alkaloid tabersonine which is a precursor of two other of our study molecules (3-oxo-tabersonine and 5-oxo-tabersonine. The alkaloids reported in this work are part of the Iboga family, which are found in various plants of the Apocynaceae family. However, the genus *Tabernaemontana* stands out for the ability of these plants to produce large amounts of this type of metabolites, as well as, the *Voacanga* genus [67,68,69].

Voacangine, voacristine and isovoacristine have shown cytotoxicity against ovarian cancer (A2780 cell line), according to our computational analyzes both have affinity with AKT1 and GSK3 proteins with averages of −9.3 kcal/mol, which showed PPIs associated with endometrial cancer signaling pathways. Cytotoxic activity of the alkaloids 3-oxocoronaridine and 5-oxocoronaridine is also reported against SKBR-3 breast adenocarcinoma and C-8161 human melanoma tumor cell lines, this may be due to the interaction of these molecules with proteins involved in the PI3K/AKT pathways such as it has been explained extensively in this study. Finally, voacangina stands out as the most evaluated molecule in carcinogenic models, giving good results in the inhibition of angiogenesis, its activity against monocytic leukemia was also reported. The alkaloid voacangine inhibits vascular endothelial growth factor receptor 2 (VEGFR2) preventing growth of glioblastoma; The affinity of voacangine with proteins such as AKT1 (−9.0 kcal/mol) and eNOS3 (−9.1 kcal/mol) can influence downstream the VEGFR2 signaling cascade [67,70].

These findings suggest the possibility of use indole type alkaloids in some controlled treatments against cellular proliferation as a consequence of its potential inhibitory effect on cancer-related molecular signaling pathways. It is know how slight changes in the molecular structure of this kind of compounds could result in activity variations [71,72]. The strongest in silico inhibitor of cellular proliferation, 5-oxocoronaridine, could give some insight concerning to the mechanism of action indole alkaloids present in *Tabernaemontana cymosa* as antitumoral agents. Therefore, it is necessary to isolate and test the biological activities of this type of molecules in different cancer cell-lines.

## 4. Materials and Methods

### 4.1. Plant Material

The plant material (seeds) was collected from the municipalities of Turbaco, Colombia. A voucher specimen was sent to the Botanical Garden William Piñeres (JBGP) staff in Cartagena, Colombia for plant identification (Voucher No. JBC 3243). The plant material was washed, dried and milled after collection. Plant material (690 g) was subjected to maceration with 2.5 L of ethanol (96%) for 3 days, with shaking intervals of 4 h. The extract obtained was filtered and the solvent was recovered under reduced pressure with a rotary evaporator at 45 °C.

### 4.2. Establishment of the Database and Protein Optimization

The crystallographic structures of 951 human proteins (ID codes are provided in Supporting Information) were obtained from the Protein Data Bank (PDB) database (National Science Foundation, Alexandria, USA). The selected proteins are involved in important physiological processes such as: Apoptosis, Cytochrome P450, Angiogenesis, Nuclear Receptors, Neuroprotection, Breast Cancer, Insulin signaling, Coagulation, Cell Proliferation, Heat Shock, Inflammation, Oxidative Stress, among others [73].

Through Sybyl-X 2.0 Suite software the proteins were prepared for molecular docking; in this process side chains were restored, ligands and water molecules were removed, additionally missing hydrogen atoms were added [74]. The resulting protein geometry was optimized using Powell’s method, Kollman United/All-atoms force fields using AMBER charges, dielectric constant 1.0, cut-off point NB 8.0, maximum number of interactions 100 and termination gradient 0.001 kcal/mol [75,76].

### 4.3. Indole Alkaloids Identification and Ligand Preparation

In previous studies carried out by our research group, Laboratory of Phytochemical and Pharmacological Research of the University of Cartagena (LIFFUC) in Cartagena, Colombia, we have identified the presence of indole alkaloids in the seeds extract of *Tabernaemontana cymosa* Jacq. and evaluated their larvicidal activity against *Aedes aegypti* L. [32,77]. The identification of these compounds from the ethanol extract of seeds of *T. cymosa* was performed using of high-performance liquid chromatography (HPLC) coupled to mass spectrometry (MS) with a Platin Blue UHPLC System (KNAVER GmbH) equipped with a Kinetex C8 Column (50 × 2.1 mm, 1.7 μm).

A reverse phase gradient elution was performed using as mobile phase mixtures of different proportions of water (Solvent A)/0.1% formic acid in methanol (Solvent B) and a flow of 350 μL/min; the gradient profile is available in supplementary data. Finally, wavelengths of 254, 290 and 332 nm were established for the detection of the eluted metabolites. The determination of the High-Resolution Mass Spectra (HRMS) was recorded on an LTQ Orbitrap (ThermoElectron-Corporation).

The 3D molecule structures chosen for the computational calculations were downloaded from the PubChem^®^ database (https://pubchem.ncbi.nlm.nih.gov/). They were subsequently optimized with the aid of Gaussian 09 program using the DFT method with B3LYP multi-parameter functional configuration. The resulting geometries were converted to the format required for molecular docking (MOL2) using the Open Babel 2.3.1 software.

Among the 23 alkaloids identified, some structural diversity was found including spiro bonds, cyclization and positional change of acyl groups, however, all molecules are derived from ibogaine alkaloids [78]. We selected the molecules 3-oxocoronaridine (CID: 14061714), 5-oxotabersonine (CID: 101850633), 3-oxotabersonine (CID: 10915101), vincinine (CID: 139595637), voacangine (CID: 73255), voacangine-7-hydroxyndolenine (CID: 328232), voacristine (CID: 196982), isovoacristina (CID: 12304498) and rupicoline (CID: 101593056) to perform the molecular docking calculations due to their structure availability in the Pubchem database. The molecule 5-oxocoronaridine (CID: C00025754) was found using KNApSAcK Core System database (http://www.knapsackfamily.com/knapsack_core/information.php?word=C00025754) [79].

### 4.4. Virtual Screening

Molecular docking calculations of indole alkaloids and 951 human targets were carried out in the AutoDock Vina 1.0 software from the Molecular Graphics Lab (La Jolla, CA, USA) at The Scripps Research Institute, the procedure included all possible binding sites using flexible docking [80]. Docking calculations for each ligand-protein complex were performed in triplicate and the best affinity values for each run constituted the average affinity energy (kcal/mol) score of indole alkaloids against human targets [81].

### 4.5. Protein–Protein Interactions (PPIs)

Protein–protein interactions (PPIs) modeling is a powerful tool to identify groups of proteins that jointly participate in signaling cascades involved in important physiological processes [82]. In this study, the STRING database (https://www.string-db.org/) was used to find physical and functional associations between the proteins with the highest affinity for indole alkaloids. A global binding affinity score was calculated for all the molecules and the three best molecules were chosen for the analysis.

First, proteins with modulation energy scores between −9.0 and −11.0 kcal/mol were selected for the construction of the network. Second, the option “multiple proteins” was configured from the STRING database and the UniProt codes of the selected complexes were entered. Finally, “Homo sapiens” was chosen as the target organism before submitting the search. In this way, protein–protein interaction networks were generated and the minimum required interaction score was established with a mean confidence of 0.4 [83,84].

### 4.6. Genomic Association

Analysis of the signaling pathways in which the target proteins participate provides more detailed information on the physiological processes affected by the molecules. The STRING database allows the search of the signaling pathways from the proteins that are part of the PPI networks through the KEGG database (https://www.kegg.jp/kegg/pathway.html). As a consequence, we obtained the probable mechanisms of action for indole alkaloids and the selection of signaling pathways was performed using the false discovery rate [85].

## 5. Conclusions

Natural products have always offered an alternative for the treatment of countless diseases. Through the computational tools applied, we were able to identify indole alkaloid targets, such as the PI3K/Akt/mTOR signaling pathway and CNS protective proteins. The 5-oxocoronaridine, voacangine-7-hydroxyindolenine and voacristine molecules presented the best theoretical affinity score with respect to human targets, highlighting those involved in carcinogenic processes, which makes them promising molecules for the development of oncological treatments; in fact, six of the indole alkaloids studied in this work have experimentally proven anticancer activity. However, the data obtained by computational techniques suggest that small structural changes in these molecules can generate significant alterations in their biological activity.

## Figures and Tables

**Table 1:** Identification of indole alkaloid type molecules present in the ethanolic extract of *T. cymosa* seeds.

Pick (N°)	Retention Time	*m/z*	Formula	Detected Molecule *	Reference
1	9.77	399.1913	C_22_H_26_O_5_N_2_	Vincinine	[35]
2	10.65	385.2122	C_22_H_28_O_4_N_2_	Rupicoline/voacangine	[36]
3	10.88	353.1858	C_21_H_24_O_3_N_2_	D-Minovincine	[37]
4	10.88	399.1913	C_22_H_26_O_5_N_2_	-	
5	11.18	385.2118	C_22_H_28_O_4_N_2_	Voacangarine	[38]
6	12.18	353.1858	C_21_H_24_O_3_N_2_	6/5/3-oxocoronaridine	[39]
7	12.45	383.1961	C_22_H_26_O_4_N_2_	19-oxovoacangine/19-iso-oxovoacangine	[40]
8	12.7	351.1701	C_21_H_22_O_3_N_2_	3/5-Oxotabersonine	[41]
9	12.97	383.1964	C_22_H_26_O_4_N_2_	19-oxovoacangine/19-iso-oxovoacangine	[40]
10	14.36	415.223	C_23_H_30_O_5_N_2_	20-hidroxyconopharyngine	[42]
11	15.44	397.1858	C_22_H_24_O_5_N_2_	-	
12	16.05	353.1856	C_21_H_24_O_3_N_2_	6/5/3-oxocoronaridine	[39]
13	16.15	383.1923	C_22_H_26_O_4_N_2_	-	
14	16.44	385.2118	C_22_H_28_O_4_N_2_	Voacristine/voacangine-7-hydroxyndolenine	[43]
15	16.6	367.2012	C_22_H_26_O_3_N_2_	Voachalotine/corymantheine	[44]
16	16.94	397.212	C_23_H_28_O_4_N_2_	-	
17	17.06	385.2118	C_22_H_28_O_4_N_2_	Voacristine/isovoacristine	[43]
18	17.27	399.2247	C_23_H_30_O_4_N_2_	Conopharyngine	[45]
19	12.64	367.2013	C_22_H_26_O_3_N_2_	Voachalotine	[44]
20	18.51	369.2179	C_22_H_28_O_3_N_2_	Voacangine/isovoacangine	[38]
21	18.9	337.1908	C_21_H_24_O_2_N_2_	Catharantine	[46]
22	18.9	369.2179	C_22_H_28_O_3_N_2_	Voacangine/isovoacangine	[38]
23	20.19	383.2326	C_23_H_30_O_3_N_2_	N-Metil-isovoacangine	[47]

* Peak identification was performed based on their retention time under established High Performance Liquid Chromatography (HPLC) conditions, High Resolution Mass Spectroscopy (HRMS) analysis and comparison with literature reports. Experimental conditions are described in supplementary material, as well as the chromatogram and spectroscopic data for isolated alkaloids.

**Table 2:** Potential targets and binding affinities of *Tabernaemontana cymosa* alkaloids to human proteins.

PDB_ ID	Predicted Binding Energy (kcal/mol)									
	5-oxocoronaridine	Voacangine-7-hydroxyndolenine	Voacristine	5-oxotabersonine	Vincinine	3-oxocoronaridine	3-oxotabersonine	Rupicoline	Isovoacristine	Voacangine
4AOJ *	−11.0	−8.0	−9.3	−8.1	−10.0	−8.9	−8.1	−8.1	−7.7	−8.0
4MJH	−10.9	−9.9	−9.0	−9.5	−9.0	−9.9	−9.8	−9.6	−9.5	−8.9
3EAH	−10.7	−8.2	−8.7	−9.1	−9.6	−8.7	−8.4	−8.7	−8.4	−9.1
4DRJ	−10.5	−9.7	−9.1	−9.3	−9.4	−9.3	−9.4	−8.9	−9.4	−8.9
4NOS	−10.4	−8.3	−9.8	−8.3	−8.4	−8.7	−8.5	−8.0	−7.8	−8.6
1N83	−10.2	−10.0	−9.1	−8.6	−9.1	−8.1	−8.8	−9.9	−10.2	−9.0
3O3U	−10.2	−9.3	−9.1	−9.5	−10.0	−8.5	−10.1	−9.0	−8.6	−8.8
4XII	−10.2	−9.2	−9.2	−9.9	−9.3	−9.6	−9.4	−10.0	−9.6	−9.4
1HFQ	−10.0	0.0	−9.2	−9.8	−9.2	−9.4	−9.9	−10.0	−9.2	−9.1
1RY8	−10.0	−9.8	−9.6	−9.9	−8.9	−8.2	−9.7	−9.0	−9.0	−9.5

* Protein information (UniProt code, name, class and biological process) is reported in Supplementary Material Table S2.

## Data Availability

PDB, KEEG PATHWAY, STRING.

## Supplementary Materials

The following are available online. Table S1: Potential targets and binding affinities of *Tabernaemontana cymosa* alkaloids to 951 human proteins evaluated; Table S2: Additional information for the 10 proteins with highest affinity score Table 2; Table S3: Node code and respective name for protein in Figure 3; Figure S1: Predicted protein interaction network for complexes with voacristine alkaloid; Figure S2. Predicted protein interaction network for complexes with voacangine-7-hydroxyndolenine alkaloid; Spectroscopic data for isolated alkaloids.

## Sample Availability

Samples of the compound voacangine are available from the authors.
