# Supplementary material for: Molecular Human Targets of Bioactive Alkaloid-Type Compounds from Tabernaemontana cymose Jacq
Source: Molecules. 2021 Jun 21;26(12):3765. doi: 10.3390/molecules26123765 (PMC8234993; doi:10.3390/molecules26123765)
Supplement: Supplementary file 1 [file molecules-26-03765-s001.zip › molecules-1247426-supplementary.pdf]

SUPPLEMENTARY MATERIAL

1. **High performance liquid chromatography and molecule identification:** The gradient profile was developed as follows: 0-1 min, 90% Solvent A; 1-3 min 80% solvent A; 3-13 min 50% solvent A; 13-24 min 33% solvent A; 24-26 min 29% solvent A; 26-35 min 25% solvent A; 35-41 min 20% solvent A; 41-45 min 2% solvent A.

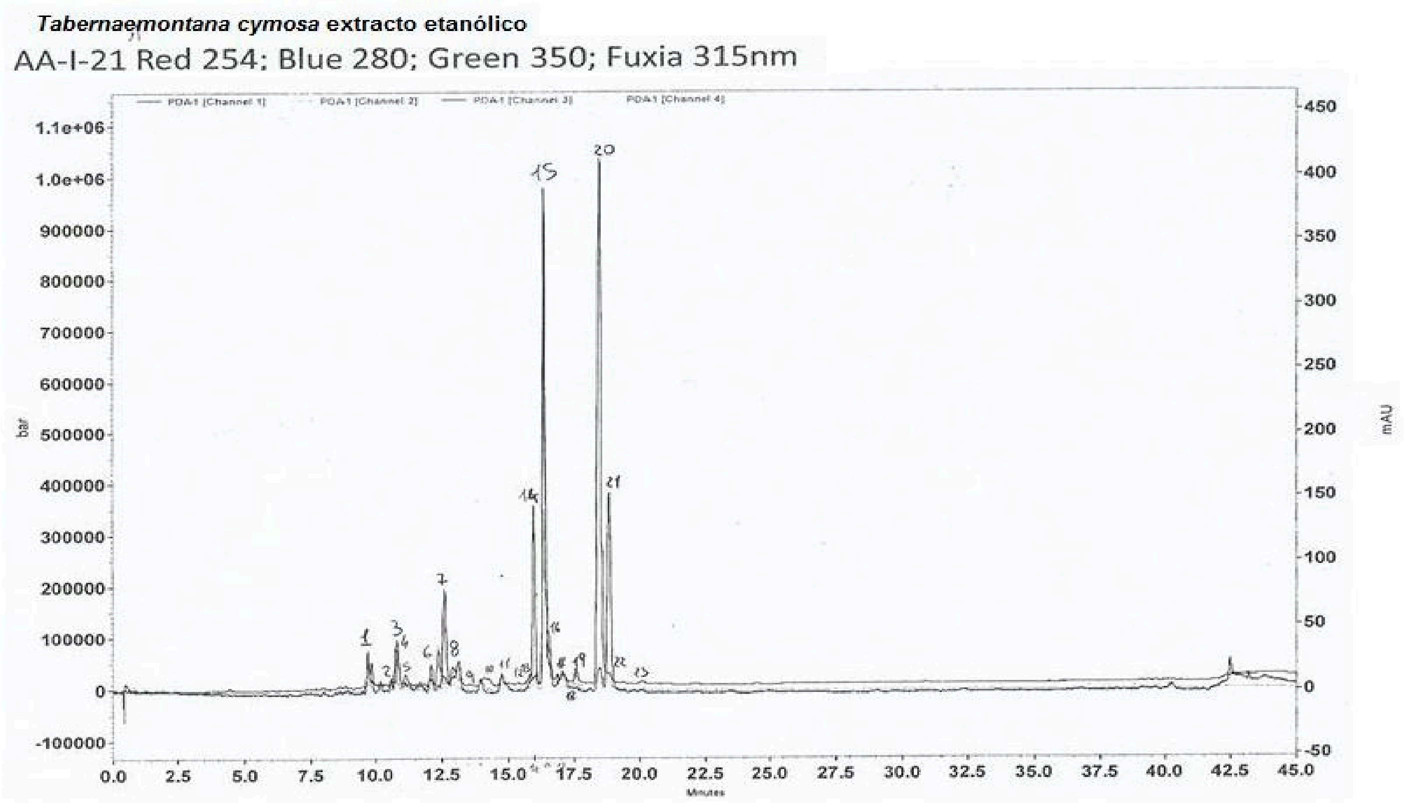

2. Molecular docking

Table S1. Potential targets and binding affinities of *Tabernaemontana cymosa* alkaloids to 951 human proteins evaluated.

	Binding affinity energies (kcal/mol)									
Protein / molecule	3-Oxo-tabersonine	3-Oxo-coronaridine	5-Oxo-coronaridine	5-Oxo-tabersonine	Iso-voacristine	Voacangine hidroxyndolenine	Rupicoline	Vincinine	Voacristine	Voacangine
**1E31**	−7.20	−6.90	−7.10	−6.60	−6.90	−6.80	−6.70	−7.40	−7.40	−7.30
**1E99**	−7.20	−8.00	−8.20	−7.00	−6.50	–	−7.00	−7.40	−7.50	−7.13
**121P**	−7.60	−8.50	−7.80	−7.50	−6.80	−8.70	−7.50	−8.00	−8.20	−8.03
**17GS**	−8.50	−8.20	−7.80	−8.60	−7.60	−7.70	−7.50	−8.10	−8.40	−7.40
**1A28**	−7.80	−7.90	−8.10	−7.70	−8.20	−8.10	−8.10	−7.90	−9.10	−8.33
**1A2B**	−7.10	−7.50	−7.60	−7.10	−6.50	−7.40	−6.80	−7.10	−7.70	−7.23
**1A4R**	−8.00	−7.90	−8.10	−8.00	−7.10	−7.90	−7.40	−7.70	−8.10	−7.40
**1A4W**	−8.50	−7.50	−7.30	−8.20	−8.40	–	−7.50	−7.00	−7.50	−8.03
**1A52**	−8.00	−7.40	−8.70	−7.50	−7.20	−7.30	−7.50	−6.80	−7.70	−7.10
**1A9U**	−7.30	−7.50	−7.80	−7.40	−6.80	–	−7.10	−7.60	−7.40	−7.37
**1AAX**	−7.10	−6.60	−7.00	−7.10	−6.80	−6.90	−7.00	−6.90	−7.10	−6.70
**1AD8**	−8.40	−7.40	−7.30	−8.20	−7.10	−7.40	−6.90	−7.10	−7.60	−7.47
**1AE8**	−8.10	−7.50	−7.80	−8.30	−7.90	–	−7.20	−7.30	−7.90	−8.47
**1AIE**	−6.90	−6.60	−6.90	−6.90	−6.10	−6.50	−6.10	−6.80	−6.40	−6.30
**1AO6**	−7.90	−8.30	−8.30	−8.10	−8.50	−8.40	−8.60	−8.00	−8.30	−8.47
**1AWI**	−7.80	−8.00	−7.70	−7.90	−7.70	−7.40	−7.10	−7.50	−8.90	−7.50

**1B1I**	−6.10	−6.90	−7.00	−6.40	−6.50	−6.70	−6.00	−6.60	−7.00	−6.47
**1B41**	−8.10	−7.50	−7.60	−8.00	−7.70	−8.10	−7.20	−7.20	−7.30	−7.20
**1BJ4**	−6.90	−7.10	−7.40	−7.50	−7.10	–	−7.20	−7.70	−7.10	−7.53
**1BP3**	−7.60	−8.10	−8.30	−7.70	−7.80	−7.80	−7.80	−7.70	−9.00	−7.70
**1BQQ**	−6.90	−7.20	−7.60	−7.00	−7.10	−7.10	−7.00	−7.40	−7.00	−7.37
**1BSX**	−7.00	−7.10	−7.00	−7.40	−8.30	−7.60	−7.20	−7.30	−7.40	−7.17
**1BX4**	−7.70	−8.10	−8.30	−8.10	−8.00	−7.80	−7.40	−8.50	−8.50	−7.60
**1C1Y**	−7.80	−7.70	−7.40	−8.00	−8.10	–	−7.50	−7.10	−7.20	−8.23
**1C5X**	−7.40	−7.20	−7.30	−7.20	−7.90	−7.60	−7.60	−7.30	−7.20	−7.77
**1C85**	−6.80	−6.60	−6.80	−6.90	−6.80	−6.90	−6.90	−6.70	−6.60	−6.53
**1CBS**	−7.30	−7.50	−8.20	−7.50	−7.10	−8.40	−8.70	−7.20	−8.10	−8.30
**1CG6**	−7.50	−7.60	−7.40	−7.90	−6.90	–	−7.20	−6.70	−7.40	−7.10
**1CJY**	−6.40	−5.80	−7.00	−7.30	−7.30	–	−6.00	−6.10	−6.30	−7.23
**1CP3**	−7.90	−7.60	−8.60	−8.20	−7.80	−7.70	−6.90	−8.00	−7.50	−7.00
**1CZA**	−7.30	−7.00	−7.20	−7.20	−7.10	−7.30	−7.30	−7.10	−7.30	−7.23
**1D5R**	−7.90	−8.10	−8.40	−8.30	−7.60	−7.90	−7.30	−7.80	−7.70	−7.60
**1DAN**	−7.70	−7.70	−7.50	−7.70	−7.40	−7.70	−7.30	−7.50	−7.80	−7.47
**1DB1**	−7.70	−8.20	−9.70	−7.20	−7.60	−8.50	−9.30	−7.20	−9.00	−9.20
**1DBH**	−7.50	−7.20	−7.80	−7.20	−7.50	−7.60	−7.80	−7.70	−7.70	−7.90
**1DCY**	−7.10	−7.50	−7.40	−7.00	−7.70	−7.60	−6.70	−7.20	−7.40	−7.97
**1DEB**	−5.80	−5.70	−6.00	−5.60	−5.50	−6.00	−5.60	−6.10	−5.90	−5.90

**1DGB**	−8.20	−8.50	−8.60	−8.80	−8.00	−8.60	−8.10	−8.30	−8.40	−7.87
**1DHT**	−8.30	−8.00	−8.60	−8.20	−8.10	–	−8.30	−8.20	−8.40	−7.80
**1DKF**	−8.30	−8.40	−9.30	−8.80	−8.30	−8.10	−7.70	−7.80	−7.90	−7.70
**1DQA**	−8.00	−7.50	−8.20	−7.80	−7.90	−7.70	−7.50	−8.10	−7.70	−7.73
**1DTW**	−7.90	−6.90	−7.20	−7.80	−8.00	−7.50	−8.30	−7.40	−7.10	−7.77
**1E3K**	−7.90	−7.90	−8.50	−7.60	−8.20	−9.50	−9.10	−8.20	−8.70	−8.33
**1E8Y**	−8.30	−8.60	−8.70	−8.10	−8.30	−8.70	−8.30	−9.00	−9.10	−8.20
**1ECV**	−6.80	−6.70	−6.90	−6.80	−6.30	−6.90	−6.80	−6.80	−7.00	−6.53
**1EDM**	−5.90	−6.20	−6.00	−6.20	−6.10	−6.00	−6.20	−6.50	−6.20	−6.60
**1EF1**	−8.60	−8.30	−8.10	−8.50	−8.20	−8.00	−8.20	−7.90	−8.50	−7.90
**1EK3**	−6.90	−7.20	−7.30	−6.80	−6.90	−7.10	−7.30	−6.90	−7.60	−7.30
**1ERR**	−7.70	−7.00	−7.90	−7.30	−7.00	−6.90	−7.70	−7.00	−6.90	−7.00
**1ETA**	−6.80	−7.40	−8.00	−6.80	−6.40	−7.10	−6.70	−7.00	−7.00	−7.30
**1ETB**	−7.20	−7.40	−7.90	−6.90	−6.40	−7.20	−6.60	−7.10	−7.20	−7.33
**1EV3**	−7.20	−7.70	−6.80	−7.20	−6.70	−6.80	−6.40	−7.20	−7.70	−6.90
**1EXT**	−7.40	−7.00	−8.30	−7.10	−7.00	−7.20	−7.90	−7.40	−7.70	−7.77
**1F3M**	−7.40	−7.10	−7.70	−7.50	−7.40	−7.20	−7.10	−7.60	−7.20	−7.17
**1F86**	−7.00	−7.50	−7.70	−7.10	−7.30	−7.60	−7.90	−7.90	−7.30	−7.20
**1F9Q**	−7.00	−6.90	−7.60	−7.60	−6.50	−7.70	−6.40	−7.50	−7.00	−7.20
**1FBY**	−7.20	−7.50	−8.50	−7.40	−7.30	−7.40	−7.80	−7.70	−7.10	−7.17
**1FCX**	−7.20	−7.30	−7.50	−7.50	−7.60	−7.00	−7.20	−8.10	−7.60	−7.37

**1FD0**	−7.20	−7.30	−8.00	−7.40	−7.60	−7.00	−7.30	−8.10	−7.70	−7.67
**1FKG**	−8.10	−7.40	−8.40	−8.10	−7.10	−7.60	−7.20	−7.80	−8.20	−7.30
**1FLT**	−7.30	−7.70	−7.20	−7.40	−7.40	−7.40	−6.80	−7.30	−7.90	−8.00
**1FM6**	−7.80	−7.90	−8.20	−8.00	−8.30	−8.20	−8.10	−7.60	−7.90	−9.20
**1FM9**	−7.20	−6.90	−7.50	−7.40	−7.30	−7.10	−6.90	−7.20	−7.40	−8.70
**1G1U**	−9.30	−8.60	−9.40	−9.10	−8.40	−8.50	−8.20	−8.80	−8.50	−8.70
**1G4U**	−6.90	−7.40	−7.80	−7.20	−6.90	–	−7.20	−7.70	−7.40	−7.80
**1G5Y**	−9.40	−8.50	−9.20	−9.20	−8.00	−8.10	−7.80	−8.90	−8.30	−8.43
**1G73**	−7.70	−7.10	−8.20	−7.50	−7.50	−8.40	−7.10	−7.40	−7.40	−7.40
**1GFW**	−8.40	−7.40	−7.20	−8.40	−7.50	−7.30	−7.10	−7.80	−7.70	−7.00
**1GH7**	−6.30	−6.30	−6.80	−6.90	−6.70	−6.60	−6.30	−6.50	−6.70	−6.40
**1GIH**	−7.40	−6.90	−7.30	−7.30	−6.80	–	−7.00	−7.90	−7.10	−7.50
**1GII**	−7.10	−7.40	−7.40	−7.30	−6.90	–	−7.00	−7.50	−7.20	−6.90
**1GJO**	−7.20	−8.30	−8.90	−7.30	−6.80	−7.60	−7.80	−7.80	−7.80	−7.60
**1GS4**	−7.30	−7.10	−7.50	−7.20	−7.00	−7.70	−6.90	−6.90	−8.30	−7.37
**1GUL**	−8.40	−7.50	−7.60	−8.10	−7.60	−7.40	−7.30	−7.40	−7.70	−7.70
**1GUM**	−8.30	−7.40	−7.10	−8.20	−7.20	−7.30	−7.30	−7.40	−7.90	−7.37
**1GWX**	−6.90	−7.10	−7.10	−7.00	−7.10	−8.20	−7.50	−6.90	−7.30	−6.73
**1H00**	−6.90	−6.70	−7.30	−7.00	−7.00	–	−7.00	−7.30	−6.90	−6.60
**1H8I**	−8.30	−7.30	−7.60	−8.20	−7.30	−7.40	−6.90	−7.30	−7.60	−7.33
**1H9O**	−6.00	−6.50	−6.20	−5.70	−5.70	−5.80	−5.90	−6.20	−6.60	−5.70

**1H9U**	−8.70	−9.40	−9.70	−8.30	−7.20	−8.30	−7.80	−8.30	−8.10	−8.90
**1HCG**	−8.30	−7.80	−7.60	−8.50	−7.20	−7.80	−6.90	−8.20	−7.50	−8.00
**1HFQ**	−9.90	−9.40	−10.00	−9.80	−9.20	–	−10.00	−9.20	−9.20	−9.10
**1HK1**	−8.20	−8.80	−8.40	−8.10	−8.20	−8.50	−8.60	−8.10	−8.20	−8.57
**1HK2**	−7.90	−8.20	−8.30	−8.40	−8.30	−8.40	−7.90	−8.30	−8.60	−8.40
**1HK3**	−8.30	−8.40	−8.40	−8.40	−8.30	−8.60	−8.30	−8.30	−8.80	−8.97
**1HK4**	−8.40	−8.20	−8.60	−8.90	−8.60	−9.00	−8.60	−8.50	−8.50	−8.13
**1HK5**	−8.90	−8.50	−9.20	−9.00	−8.30	−8.60	−8.60	−8.90	−8.60	−7.93
**1I09**	−7.90	−7.80	−8.80	−7.60	−7.20	−7.50	−7.70	−7.80	−8.00	−7.50
**1I3O**	−7.30	−7.20	−8.10	−7.50	−7.40	−7.50	−7.30	−7.80	−7.70	−7.67
**1I44**	−6.70	−6.50	−6.50	−6.40	−6.30	−6.30	−6.10	−6.60	−6.90	−6.30
**1I5R**	−8.80	−8.00	−9.40	−8.60	−7.50	−8.60	−7.40	−9.50	−8.30	−7.77
**1I7I**	−7.90	−8.00	−8.40	−7.70	−7.40	−7.90	−7.20	−7.90	−8.20	−7.90
**1ICT**	−7.90	−8.00	−8.20	−8.00	−7.90	−8.70	−8.40	−8.20	−8.30	−8.20
**1IG1**	−9.00	−7.80	−8.10	−9.00	−7.60	−8.20	−7.80	−8.00	−8.00	−7.60
**1IMV**	−7.70	−7.10	−7.20	−7.60	−7.00	−7.10	−6.80	−7.40	−7.40	−6.80
**1IR3**	−6.60	−7.50	−7.70	−6.80	−7.10	−7.50	−6.40	−7.50	−7.00	−7.00
**1IT6**	−8.60	−7.30	−7.50	−8.60	−7.90	−7.50	−7.20	−7.50	−7.00	−7.20
**1J1A**	−8.50	−8.80	−9.20	−9.10	−8.10	−9.00	−8.70	−9.10	−8.60	−8.13
**1J1B**	−7.80	−8.50	−8.20	−7.50	−7.50	–	−7.60	−9.00	−7.90	−7.57
**1J4X**	−6.40	−6.20	−6.20	−6.40	−6.60	−6.00	−6.20	−6.20	−6.10	−5.90

**1J8F**	−8.60	−8.30	−8.50	−8.40	−7.90	−8.10	−8.20	−8.10	−8.50	−8.30
**1JDH**	−7.20	−6.70	−6.80	−6.90	−6.60	−6.60	−6.40	−7.10	−6.70	−7.00
**1JF7**	−7.40	−7.50	−7.40	−7.40	−7.20	−8.00	−7.40	−7.40	−7.70	−7.57
**1JKL**	−9.10	−7.70	−8.00	−9.20	−7.80	−7.70	−7.70	−7.60	−8.00	−7.80
**1JMJ**	−7.70	−8.20	−7.90	−8.00	−7.90	−7.70	−7.50	−8.40	−7.50	−7.17
**1JSV**	−7.20	−7.70	−8.30	−6.90	−6.90	–	−6.70	−8.20	−7.30	−7.63
**1JU5**	−6.20	−6.50	−7.40	−6.60	−6.70	−7.00	−7.20	−7.20	−7.20	−7.10
**1JYR**	−6.00	−5.80	−6.00	−6.30	−6.10	−5.90	−6.30	−6.20	−6.00	−5.93
**1K3A**	−7.40	−7.30	−7.50	−7.50	−7.50	−7.40	−7.00	−7.90	−7.50	−7.17
**1K6M**	−7.50	−8.70	−7.90	−8.10	−7.30	−8.40	−9.50	−7.50	−8.90	−7.33
**1K74**	−7.30	−6.90	−7.70	−7.40	−7.00	−6.80	−6.70	−6.90	−7.00	−6.93
**1K7L**	−8.10	−7.80	−7.50	−8.10	−7.90	−7.90	−7.40	−7.60	−7.90	−7.37
**1KAO**	−7.10	−6.80	−7.60	−7.30	−6.90	–	−7.00	−7.30	−6.80	−6.90
**1KAV**	−6.70	−6.50	−6.90	−6.70	−6.90	−7.10	−7.00	−6.70	−6.80	−6.80
**1KBH**	−6.40	−6.30	−6.50	−6.40	−6.60	−6.10	−6.00	−6.10	−6.30	−6.50
**1KE5**	−7.10	−7.10	−7.60	−6.90	−6.40	–	−7.30	−7.70	−7.10	−7.00
**1KE8**	−7.10	−7.30	−7.80	−6.90	−6.50	–	−7.30	−7.70	−7.30	−7.10
**1KKQ**	−7.70	−7.50	−8.40	−8.20	−8.20	−8.20	−8.60	−8.00	−8.00	−8.30
**1KPF**	−6.80	−7.30	−7.70	−7.00	−6.90	−6.80	−6.80	−6.70	−7.30	−7.00
**1KV2**	−7.60	−8.70	−8.50	−7.60	−7.60	−7.80	−7.40	−8.60	−8.00	−7.63
**1KV6**	−7.70	−7.30	−8.40	−7.50	−7.30	−7.60	−7.80	−7.00	−7.40	−8.00

**1L6J**	−8.00	−7.50	−8.40	−8.10	−7.80	−8.30	−7.40	−7.60	−8.00	−7.20
**1LBD**	−6.90	−8.10	−7.20	−6.80	−7.00	−7.70	−7.00	−6.80	−8.40	−6.80
**1LV2**	−7.50	−7.60	−8.10	−8.00	−7.50	−8.60	−7.80	−7.50	−8.30	−7.60
**1LYW**	−8.50	−8.20	−8.90	−8.90	−7.60	−8.70	−8.10	−8.20	−8.40	−9.47
**1M05**	−7.90	−9.10	−9.20	−8.10	−8.90	−8.60	−8.80	−8.60	−8.90	−8.07
**1M13**	−8.40	−8.90	−9.60	−8.60	−8.70	−8.80	−8.50	−8.30	−8.50	−8.43
**1M17**	−7.80	−7.40	−7.70	−7.80	−7.70	−7.40	−7.00	−7.60	−7.80	−7.37
**1MAZ**	−7.60	−7.30	−7.10	−7.30	−7.00	−7.20	−6.90	−6.90	−7.30	−7.70
**1MCH**	−7.50	−7.70	−7.90	−7.70	−8.00	–	−7.80	−8.30	−7.70	−7.60
**1MQ4**	−7.80	−8.50	−8.20	−8.20	−6.80	−8.50	−7.10	−9.50	−8.60	−7.17
**1MSO**	−5.90	−6.00	−6.30	−6.30	−6.00	−5.90	−5.80	−6.60	−6.20	−6.30
**1MV9**	−7.80	−6.90	−7.90	−7.20	−6.80	−7.10	−7.30	−8.20	−7.20	−7.20
**1MVC**	−7.70	−6.80	−7.90	−7.20	−7.20	−7.00	−6.60	−8.30	−6.90	−7.20
**1MZN**	−6.90	−7.60	−8.20	−6.90	−7.70	−6.50	−6.90	−7.40	−7.40	−6.70
**1N4Q**	−7.40	−7.40	−8.00	−7.90	−8.00	–	−8.30	−7.70	−7.60	0.00
**1N83**	−8.80	−8.10	−10.20	−8.60	−10.20	−10.00	−9.90	−9.10	−9.10	−9.03
**1NAX**	−7.30	−8.80	−8.10	−7.40	−7.60	−7.70	−7.10	−7.20	−7.90	−8.00
**1NCF**	−7.00	−7.00	−7.00	−6.90	−7.10	–	−6.70	−7.70	−7.30	−7.20
**1NFI**	−7.60	−7.00	−8.10	−8.00	−7.30	–	−7.20	−7.60	−7.60	−7.57
**1NME**	−7.20	−7.20	−7.40	−7.20	−7.00	−6.80	−6.80	−7.00	−7.10	−6.83
**1NMQ**	−8.20	−7.80	−9.30	−8.60	−7.10	−8.00	−7.20	−8.00	−8.00	−7.23

**1NMS**	−7.80	−8.00	−8.20	−8.10	−8.30	−8.50	−7.70	−8.10	−8.10	−7.60
**1NQL**	−7.50	−6.90	−7.50	−7.70	−7.00	–	−6.80	−7.30	−6.80	−6.70
**1NRL**	−8.40	−8.30	−8.80	−8.20	−8.20	−8.50	−8.70	−7.60	−7.90	−8.40
**1NSI**	−7.80	−8.40	−9.70	−8.30	−7.60	–	−7.60	−8.50	−8.70	−8.63
**1NW9**	−6.80	−6.80	−7.30	−6.80	−7.00	−6.60	−6.30	−6.80	−7.00	−6.43
**1O8A**	−8.90	−8.90	−9.20	−9.10	−9.00	−9.20	−9.00	−9.20	−9.20	−8.70
**1OI9**	−7.70	−8.00	−9.40	−7.80	−7.70	−8.60	−7.80	−8.00	−7.90	−7.73
**1OJ9**	−7.70	−7.50	−7.70	−8.00	−7.50	−7.70	−7.20	−7.50	−7.60	−7.30
**1OJA**	−7.90	−7.80	−8.10	−7.60	−8.20	−8.20	−7.90	−7.60	−8.40	−7.53
**1OJC**	−8.00	−8.50	−7.80	−8.30	−7.60	−7.90	−7.80	−7.70	−8.30	−7.57
**1OL5**	−6.90	−8.50	−8.80	−7.60	−7.80	–	−7.70	−8.70	−9.40	−7.80
**1OL6**	−7.70	−8.10	−8.30	−7.50	−7.60	–	−8.00	−9.00	−8.40	−7.60
**1OSF**	−7.80	−7.50	−7.60	−7.10	−7.10	−7.40	−7.10	−7.20	−7.60	−7.30
**1OVL**	−8.30	−7.90	−8.30	−8.30	−7.70	−8.40	−8.10	−8.70	−8.50	−7.97
**1P0I**	−9.80	−9.60	−10.10	−9.90	−9.30	−9.00	−8.70	−9.10	−9.40	−9.70
**1P22**	−6.70	−7.20	−7.20	−6.90	−6.80	−6.90	−6.60	−7.50	−7.30	−6.90
**1P2S**	−7.50	−7.50	−7.90	−7.80	−7.70	−7.30	−7.40	−7.70	−7.50	−7.67
**1P8D**	−8.50	−9.20	−9.50	−8.60	−7.80	−11.00	−9.30	−8.90	−8.80	−8.37
**1P93**	−8.10	−7.30	−8.80	−8.20	−7.80	−8.70	−8.60	−8.00	−8.30	−7.57
**1PAU**	−6.70	−6.80	−6.80	−6.60	−6.20	−6.90	−6.20	−6.60	−6.90	−6.50
**1PQ6**	−8.00	−8.50	−8.90	−8.20	−7.90	−8.20	−7.90	−8.60	−8.50	−7.80

**1PY5**	−7.50	−6.90	−7.10	−6.70	−6.60	−6.90	−7.20	−7.20	−7.90	−6.90
**1Q5K**	−7.70	−8.40	−8.30	−7.70	−7.60	–	−7.30	−8.90	−8.20	−7.77
**1QDU**	−7.20	−6.60	−7.80	−7.40	−7.30	−8.20	−7.30	−7.10	−7.30	−7.37
**1QKU**	−7.60	−7.60	−8.30	−7.70	−7.70	−8.20	−7.60	−7.40	−7.70	−7.80
**1QQG**	−6.90	−8.00	−8.30	−7.20	−7.60	−8.60	−7.40	−7.50	−9.20	−7.20
**1QXK**	−7.70	−6.30	−6.70	−7.20	−6.90	−6.80	−6.80	−7.10	−6.80	−6.80
**1R2D**	−7.40	−7.40	−7.90	−7.40	−6.80	−7.10	−6.90	−6.90	−7.10	−7.03
**1RE1**	−7.40	−6.70	−7.20	−7.20	−6.90	−6.90	−6.80	−7.00	−7.10	−6.60
**1RGP**	−7.30	−7.10	−7.10	−7.50	−6.40	−6.60	−6.20	−6.70	−6.90	−6.60
**1RWW**	−7.70	−6.90	−7.60	−7.50	−7.00	−7.40	−7.00	−7.20	−6.90	−7.10
**1RY8**	−9.70	−8.20	−10.00	−9.90	−9.00	−9.80	−9.00	−8.90	−9.60	−9.53
**1S2Q**	−7.50	−7.80	−7.90	−7.50	−7.60	−7.80	−8.00	−7.40	−7.70	−7.50
**1S2Y**	−7.90	−8.10	−8.10	−8.20	−7.80	−8.10	−8.00	−7.80	−8.10	−7.67
**1S3B**	−7.80	−7.90	−8.10	−8.10	−7.80	−8.10	−7.80	−7.80	−8.20	−7.80
**1S3E**	−7.70	−8.10	−8.00	−7.80	−7.80	−8.00	−7.90	−7.30	−8.10	−7.50
**1S3X**	−6.70	−7.20	−8.30	−6.60	−6.90	−7.90	−7.80	−6.80	−7.50	−7.00
**1S70**	−8.50	−7.70	−8.50	−8.20	−7.60	−8.30	−7.60	−8.80	−7.80	−7.73
**1S9V**	0.00	0.00	0.00	0.00	0.00	0.00	0.00	0.00	0.00	0.00
**1SAC**	−7.20	−7.00	−7.30	−7.00	−6.90	−7.20	−7.10	−7.70	−7.50	−7.00
**1SJ0**	−7.40	−6.80	−7.10	−7.70	−7.00	−6.70	−6.70	−6.90	−7.20	−7.53
**1SM2**	−7.40	−7.80	−7.80	−7.20	−7.60	–	−7.30	−7.90	−7.50	−6.87

**1SQN**	−7.30	−9.00	−8.60	−7.40	−8.30	−7.70	−7.70	−8.30	−8.80	−7.93
**1SR7**	−7.80	−8.10	−8.90	−7.20	−7.80	−8.40	−8.90	−6.90	−8.30	−8.23
**1SVC**	−6.50	−6.50	−6.90	−6.50	−6.30	−7.00	−6.20	−6.80	−6.50	−6.20
**1T64**	−7.40	−7.60	−7.90	−7.20	−7.30	−7.50	−7.20	−7.50	−7.60	−7.80
**1T67**	−6.60	−6.40	−6.70	−6.60	−6.50	−6.70	−6.90	−7.20	−6.50	−6.97
**1T69**	−6.50	−6.60	−6.70	−6.40	−6.90	−6.60	−6.50	−6.80	−6.70	−6.53
**1T7H**	−5.20	−5.50	−5.50	−5.40	−5.10	−5.20	−5.00	−5.60	−5.80	−5.40
**1TB6**	−8.50	−7.70	−9.10	−7.80	−8.00	−7.70	−8.20	−7.60	−8.00	−7.97
**1THA**	−6.20	−6.00	−6.30	−6.20	−5.60	−6.40	−5.70	−5.90	−6.40	−6.00
**1TNF**	−7.00	−7.60	−7.20	−7.00	−7.80	−8.60	−8.00	−6.70	−7.60	−8.00
**1TOU**	−9.70	−9.20	−8.50	−9.90	−8.00	−8.80	−8.30	−9.00	−8.40	−8.10
**1TQN**	−8.70	−9.50	−8.90	−8.60	−8.60	−9.10	−8.40	−9.40	−9.90	−8.60
**1TW6**	−6.80	−7.40	−7.20	−6.90	−6.80	−7.80	−7.30	−6.90	−7.00	−7.00
**1U4D**	−7.60	−8.60	−8.50	−7.50	−7.20	−8.00	−7.90	−9.30	−8.10	−7.33
**1UHL**	−8.90	−8.10	−9.90	−8.70	−8.30	−9.00	−8.70	−8.30	−8.50	−8.90
**1UNL**	−6.80	−7.50	−9.10	−6.80	−6.70	−7.90	−7.00	−7.20	−7.70	−6.93
**1UOM**	−7.30	−6.90	−8.00	−7.00	−6.60	−7.00	−6.40	−7.20	−7.10	−7.47
**1UOU**	−6.50	−7.30	−6.80	−6.60	−6.40	−7.20	−6.90	−6.50	−6.90	−6.83
**1UPK**	−7.10	−7.10	−7.20	−6.90	−6.70	–	−6.60	−6.80	−7.00	−7.10
**1US0**	−7.90	−7.70	−8.90	−7.90	−7.80	−7.30	−7.40	−7.30	−8.00	−7.50
**1UV5**	−7.40	−8.60	−8.50	−7.30	−7.00	−7.40	−6.90	−9.60	−8.90	−7.40

**1UY6**	−8.80	−9.70	−9.20	−8.80	−7.90	−7.60	−7.60	−7.80	−9.10	−7.73
**1UY7**	−8.90	−7.70	−9.70	−9.00	−8.10	−8.10	−7.70	−8.10	−10.10	−7.93
**1UY8**	−8.80	−8.60	−9.20	−9.00	−7.90	−7.30	−7.50	−8.00	−9.60	−7.93
**1UY9**	−8.90	−10.00	−9.40	−9.00	−7.90	−7.10	−7.70	−7.90	−9.80	−7.83
**1UYC**	−8.80	−9.90	−9.50	−9.00	−7.60	−7.10	−7.60	−7.90	−9.70	−8.00
**1UYD**	−8.80	−9.70	−9.30	−9.00	−7.60	−8.00	−7.60	−7.80	−8.30	−7.67
**1UYE**	−8.90	−9.30	−9.30	−8.90	−7.40	−8.10	−7.60	−7.60	−9.80	−7.70
**1UYF**	−8.80	−7.80	−9.20	−9.00	−7.50	−8.00	−7.50	−7.70	−9.00	−7.63
**1UYG**	−9.10	−8.00	−8.50	−9.30	−7.30	−7.10	−7.30	−7.50	−8.10	−7.60
**1UYH**	−8.40	−7.80	−8.90	−9.00	−7.30	−7.00	−7.20	−8.10	−8.60	−7.70
**1UYI**	−8.80	−7.80	−8.80	−8.80	−7.20	−7.30	−7.50	−7.30	−7.80	−7.90
**1UYK**	−8.90	−8.00	−9.20	−9.00	−7.60	−8.00	−7.40	−8.00	−9.30	−7.67
**1UYM**	−8.60	−9.50	−9.00	−8.60	−7.50	−8.10	−7.40	−7.80	−9.60	−7.63
**1V04**	−8.80	−8.80	−8.50	−8.70	−9.40	−10.10	−9.50	−8.60	−8.70	−8.53
**1V4S**	−8.50	−8.10	−8.40	−8.50	−8.40	−8.20	−7.70	−7.60	−8.20	−8.20
**1VGE**	−7.30	−7.30	−7.70	−7.60	−7.80	−7.00	−7.40	−7.20	−7.50	−7.50
**1W22**	−8.20	−7.30	−8.70	−7.70	−8.20	−7.60	−7.00	−7.60	−8.00	−8.67
**1WBN**	−8.20	−8.20	−8.20	−8.20	−8.20	−8.30	−7.80	−8.20	−8.30	−7.93
**1WDZ**	−7.80	−7.40	−7.60	−7.90	−6.70	−7.00	−7.80	−7.40	−7.50	−6.90
**1WKW**	−7.70	−8.70	−9.00	−8.00	−7.40	−7.50	−7.10	−7.50	−8.20	−8.73
**1X78**	−7.20	−7.50	−8.50	−7.00	−7.10	−7.10	−7.40	−7.50	−7.90	−7.10

**1X7E**	−9.00	−7.80	−8.70	−9.70	−7.80	−8.40	−8.10	−7.60	−8.20	−8.37
**1XAP**	−7.50	−7.00	−7.20	−7.30	−7.20	−6.80	−6.50	−7.50	−7.80	−7.10
**1XB7**	−7.10	−7.00	−6.60	−6.80	−7.30	−7.10	−6.60	−6.30	−7.00	−6.97
**1XJD**	−7.00	−8.40	−7.80	−6.90	−6.80	−7.80	−7.60	−9.10	−8.00	−7.10
**1XTS**	−8.00	−7.90	−8.30	−8.10	−6.40	−6.70	−7.00	−7.50	−6.50	−6.80
**1XZX**	−7.70	−7.80	−7.50	−7.40	−7.20	−7.80	−7.10	−8.10	−7.80	−7.50
**1Y0X**	−7.30	−7.10	−7.60	−7.30	−7.30	−7.70	−7.00	−7.30	−7.30	−7.67
**1Y2O**	−7.60	−7.20	−7.60	−7.90	−7.10	−7.60	−7.80	−7.40	−7.10	−7.70
**1Y57**	−7.30	−7.60	−7.80	−7.60	−7.60	−7.70	−6.80	−8.20	−7.40	−7.13
**1YC3**	−7.60	−7.60	−7.50	−7.60	−6.80	−7.60	−6.40	−6.50	−6.60	−7.03
**1YET**	−7.50	−7.60	−7.30	−7.80	−7.10	−7.20	−7.00	−7.60	−7.40	−7.10
**1YK1**	−8.10	−7.90	−8.70	−8.10	−8.60	−7.90	−7.60	−8.20	−8.70	−7.83
**1YOK**	−9.00	−8.80	−8.70	−9.30	−8.80	−8.30	−8.50	−9.10	−8.20	−8.70
**1YRP**	−9.20	−8.40	−9.10	−9.30	−9.10	−8.80	−8.70	−8.60	−8.90	−9.00
**1YTV**	−9.50	−9.90	−9.80	−9.40	−8.50	−9.30	−9.00	−9.90	−8.90	−8.77
**1YWN**	−7.30	−6.90	−7.60	−6.90	−6.90	−7.30	−7.30	−7.50	−6.80	−7.10
**1YWT**	−7.80	−7.40	−7.30	−7.80	−7.40	−7.20	−7.20	−7.40	−7.70	−7.30
**1Z2C**	−7.00	−7.30	−7.60	−7.20	−7.20	−7.60	−7.20	−7.40	−6.90	−7.73
**1Z57**	−7.60	−8.10	−9.80	−7.60	−7.30	–	−7.70	−9.50	−9.00	−7.13
**1Z6T**	−8.00	−8.10	−9.00	−8.80	−8.00	−9.20	−8.30	−8.00	−8.40	−8.20
**1Z7J**	−6.40	−7.30	−7.20	−6.80	−6.20	−7.00	−6.70	−6.90	−7.10	−6.80

**1ZGY**	−7.30	−8.50	−8.90	−8.70	−8.40	−8.50	−7.60	−9.10	−8.70	−9.00
**1ZHM**	−8.20	−7.40	−7.70	−8.30	−7.10	−7.70	−7.60	−7.40	−7.50	−6.93
**1ZUC**	−7.70	−7.70	−7.90	−7.70	−8.60	−7.50	−7.90	−7.40	−8.40	−8.17
**1ZUQ**	−7.00	−6.40	−7.00	−6.90	−6.80	−6.60	−6.60	−6.90	−6.50	−6.80
**1ZXC**	−7.70	−7.20	−8.50	−7.40	−7.20	–	−8.20	−7.80	−7.50	−8.07
**1ZXM**	−8.30	−8.20	−9.60	−9.00	−8.70	−8.60	−9.30	−8.70	−8.70	−8.60
**1ZYJ**	−7.50	−7.60	−7.80	−7.70	−7.40	−7.30	−7.30	−8.30	−7.80	−7.50
**1ZYS**	−7.20	−7.80	−7.80	−7.10	−7.40	–	−7.40	−7.30	−7.70	−7.50
**2A19**	−7.80	−7.80	−8.60	−7.80	−7.50	−7.30	−7.50	−8.20	−7.90	−7.53
**2A1A**	−7.70	−7.20	−7.90	−7.80	−7.50	−7.50	−7.70	−7.80	−7.60	−7.70
**2ATX**	−7.00	−6.80	−7.00	−6.80	−6.80	−7.00	−6.80	−6.90	−7.10	−6.77
**2AZ5**	−8.70	−8.10	−8.60	−8.80	−7.60	–	−7.90	−8.30	−8.20	−7.97
**2B0Y**	−5.60	−5.80	−5.60	−5.70	−5.40	−6.30	−5.20	−6.10	−5.30	−5.70
**2B7A**	−6.70	−6.80	−7.50	−7.00	−7.70	–	−7.70	−7.00	−7.30	−7.13
**2BAW**	−7.90	−7.30	−8.10	−8.30	−7.70	−7.50	−8.30	−7.80	−7.80	−7.23
**2BEL**	−8.70	−8.80	−9.30	−9.10	−9.30	−9.20	−8.90	−9.10	−9.10	−9.00
**2BGD**	−6.60	−6.70	−7.10	−6.80	−6.60	−6.80	−6.90	−6.70	−6.60	−6.80
**2BGE**	−6.90	−6.70	−6.60	−7.00	−6.70	−6.90	−6.30	−6.80	−6.60	−6.50
**2BIT**	−6.20	−6.40	−6.60	−6.30	−6.10	−7.10	−6.70	−6.30	−6.20	−6.43
**2BK3**	−7.60	−7.70	−7.80	−7.50	−7.60	−7.90	−7.90	−7.80	−8.00	−7.50
**2BOH**	−8.00	−8.40	−7.90	−8.40	−7.40	−7.50	−7.10	−7.70	−7.30	−8.33

**2BSM**	−6.80	−6.20	−7.60	−6.90	−6.20	−6.60	−5.80	−6.30	−6.40	−6.33
**2BT0**	−8.20	−8.00	−8.00	−7.80	−7.80	−8.20	−7.70	−8.30	−8.00	−8.17
**2BUJ**	−7.60	−7.40	−7.30	−7.50	−7.10	−7.30	−7.10	−7.90	−7.50	−7.70
**2BVR**	−7.70	−7.70	−7.20	−7.80	−8.50	−7.60	−7.40	−7.10	−7.70	−7.90
**2BYB**	−7.70	−7.80	−8.20	−8.00	−7.70	−8.00	−7.50	−8.00	−8.00	−7.53
**2BYH**	−7.20	−7.10	−7.40	−7.50	−6.80	−6.90	−6.50	−7.40	−6.70	−6.63
**2BYI**	−7.30	−7.20	−7.40	−7.20	−6.70	−8.20	−7.00	−7.30	−7.80	−7.13
**2BZ5**	−7.80	−7.50	−7.60	−7.60	−7.30	−7.80	−7.60	−8.20	−7.90	−7.67
**2C64**	−7.60	−7.80	−8.10	−7.80	−7.40	−7.60	−7.80	−7.50	−7.90	−7.70
**2C65**	−7.40	−7.90	−8.10	−8.10	−7.80	−8.00	−7.90	−7.70	−8.10	−7.57
**2C66**	−7.40	−7.70	−8.00	−7.80	−7.80	−7.90	−7.60	−7.80	−7.80	−7.57
**2C67**	−7.90	−7.40	−8.00	−7.70	−7.80	−7.90	−7.80	−7.80	−7.70	−7.57
**2C70**	−7.80	−7.40	−8.00	−8.00	−7.50	−7.90	−7.80	−7.60	−7.80	−7.60
**2CBZ**	−7.20	−6.80	−7.50	−7.60	−7.10	−6.60	−7.50	−6.80	−7.10	−6.80
**2CCS**	−6.80	−6.30	−7.60	−6.90	−6.30	−6.70	−6.10	−6.50	−6.50	−6.10
**2CCT**	−6.90	−6.40	−7.30	−6.90	−6.30	−6.80	−6.20	−6.50	−6.60	−6.43
**2CCU**	−6.70	−6.60	−6.70	−6.80	−6.30	−6.60	−6.40	−6.50	−7.00	−6.63
**2CEO**	−7.30	−7.80	−7.40	−7.30	−7.70	−7.40	−7.40	−7.60	−7.60	−7.80
**2CKE**	−8.20	−7.90	−8.50	−8.30	−8.30	−8.40	−8.30	−7.90	−8.70	−8.53
**2D0T**	−8.00	−8.10	−8.90	−8.00	−7.90	−8.20	−8.40	−8.10	−8.20	−8.40
**2DUX**	−7.70	−7.80	−8.00	−7.70	−7.60	−7.50	−6.90	−7.40	−7.90	−8.00

**2E0T**	−6.40	−6.60	−6.50	−6.40	−5.90	−6.30	−5.70	−6.40	−6.70	−6.40
**2EB3**	−7.00	−6.90	−7.50	−7.00	−7.60	–	−7.00	−7.60	−7.10	−7.60
**2EFK**	−6.50	−6.40	−6.40	−6.70	−6.00	−6.50	−6.10	−6.30	−6.90	−6.27
**2EVW**	−7.00	−6.50	−7.10	−7.00	−7.10	−6.80	−6.50	−7.30	−6.90	−7.50
**2F70**	−7.40	−6.70	−6.90	−7.30	−6.80	−6.80	−7.30	−6.50	−7.00	−6.70
**2F71**	−7.10	−6.70	−6.90	−7.00	−6.60	−6.90	−6.60	−6.70	−6.90	−6.63
**2FDA**	−7.90	−7.60	−7.90	−8.10	−7.00	−7.30	−7.10	−7.80	−7.80	−7.17
**2FDV**	−8.00	−7.70	−8.20	−7.70	−7.40	−8.00	−7.70	−7.90	−8.20	−7.40
**2FJ5**	−5.00	−5.10	−5.60	−5.20	−5.40	−5.40	−4.80	−5.40	−5.30	−5.27
**2FV5**	−7.10	−7.50	−8.00	−7.30	−7.90	–	−7.60	−8.00	−8.30	−8.13
**2FWY**	−9.00	−8.90	−9.50	−8.90	−7.90	−7.30	−7.80	−8.00	−8.40	−7.80
**2FWZ**	−8.50	−9.40	−9.40	−8.60	−7.70	−7.10	−7.90	−7.70	−9.70	−8.00
**2G0H**	−7.60	−7.80	−8.80	−7.80	−7.50	−7.90	−6.90	−8.20	−7.90	−7.60
**2GCP**	−6.80	−7.60	−8.00	−7.10	−6.50	−6.50	−6.40	−6.90	−6.90	−7.13
**2GL8**	−8.30	−8.30	−8.70	−8.60	−8.40	−8.10	−8.60	−8.00	−8.10	−8.37
**2GS2**	−8.00	−7.40	−8.10	−8.00	−7.70	–	−7.10	−7.70	−7.00	−7.30
**2GS6**	−8.20	−7.70	−8.20	−8.40	−7.70	−7.80	−7.30	−8.10	−7.70	−7.57
**2H55**	−8.80	−7.90	−9.10	−8.60	−8.00	−7.50	−7.10	−7.80	−9.60	−7.80
**2H77**	−8.70	−7.40	−7.40	−8.80	−7.60	−6.90	−6.70	−7.50	−7.70	−7.90
**2H79**	−8.20	−7.80	−8.10	−8.30	−7.50	−7.40	−7.20	−8.00	−7.50	−7.30
**2H7C**	−9.00	−8.70	−8.70	−8.60	−8.60	−8.80	−8.90	−8.50	−9.00	−8.50

**2H94**	−8.70	−6.70	−8.10	−8.20	−7.50	−9.20	−8.40	−7.30	−7.60	−8.00
**2HI4**	−8.00	−7.40	−8.10	−7.90	−7.70	−7.70	−7.20	−7.40	−7.60	−7.57
**2HK5**	−7.50	−7.70	−8.10	−7.50	−7.50	−6.90	−6.60	−7.80	−8.30	−7.23
**2HKK**	−7.30	−7.30	−7.50	−7.50	−6.90	−7.40	−6.60	−7.00	−7.10	−7.27
**2HMH**	−8.20	−7.70	−7.90	−8.60	−7.70	−7.30	−7.30	−7.90	−7.20	−7.70
**2HR7**	−7.70	−7.10	−7.90	−8.90	−7.20	−7.50	−7.10	−7.40	−7.30	−7.43
**2HWQ**	−7.40	−8.00	−8.50	−7.70	−7.90	−8.50	−8.50	−7.60	−8.00	−7.67
**2HXL**	−7.40	−7.50	−7.50	−7.90	−7.70	−8.00	−7.80	−7.20	−8.20	−7.90
**2I47**	−7.30	−7.30	−8.10	−7.10	−7.10	–	−7.50	−7.70	−7.60	−7.53
**2ITN**	−7.20	−7.50	−8.30	−7.90	−7.40	–	−7.20	−7.70	−8.20	−7.50
**2ITP**	−7.20	−8.20	−8.20	−7.50	−7.50	–	−7.20	−8.40	−8.60	−7.63
**2ITU**	−7.40	−7.40	−8.00	−7.70	−6.90	–	−7.00	−7.80	−7.70	−7.10
**2ITW**	−8.70	−8.20	−8.60	−8.50	−7.90	–	−7.10	−8.10	−8.10	−7.60
**2ITX**	−7.20	−7.30	−7.60	−7.80	−7.20	–	−7.10	−7.60	−7.60	−6.93
**2ITZ**	−7.00	−7.60	−8.30	−7.20	−7.00	–	−6.80	−7.60	−7.90	−6.80
**2J14**	−8.10	−7.80	−8.10	−8.00	−7.90	−7.80	−7.50	−7.70	−7.80	−7.30
**2J3N**	−8.30	−7.80	−9.10	−8.40	−8.30	−8.80	−8.70	−8.00	−7.80	−8.67
**2J4I**	−8.60	−8.00	−7.80	−8.60	−7.80	−7.80	−7.10	−7.40	−7.40	−8.20
**2J8Z**	−7.00	−6.50	−6.60	−6.60	−6.80	−6.80	−6.30	−6.70	−6.50	−6.20
**2JDF**	−7.10	−6.90	−7.10	−7.20	−6.80	−6.90	−7.10	−7.20	−6.60	−6.20
**2JF9**	−7.40	−7.20	−7.80	−7.30	−6.80	−7.30	−7.20	−7.10	−7.00	−7.27

**2JFA**	−7.90	−6.80	−7.20	−8.20	−7.80	−7.00	−7.70	−7.70	−6.80	−8.57
**2JJC**	−6.90	−6.30	−6.90	−7.10	−6.20	−6.90	−6.40	−6.40	−6.60	−6.40
**2JJK**	−7.60	−7.90	−8.40	−8.20	−8.20	−7.60	−7.40	−7.80	−8.10	−7.83
**2JLP**	−8.80	−7.80	−8.10	−7.70	−8.30	−8.00	−8.20	−7.90	−8.00	−7.63
**2JQ6**	−6.70	−6.60	−6.80	−6.40	−6.30	−6.30	−6.40	−6.70	−6.60	−6.30
**2KAV**	−6.50	−6.80	−7.00	−7.00	−6.30	−6.10	−5.90	−6.50	−6.60	−7.30
**2LDC**	−3.60	−3.80	−3.90	−3.70	−4.20	−4.10	−3.70	−3.60	−4.20	−3.70
**2LWI**	−6.90	−6.90	−7.10	−6.60	−7.10	–	−6.90	−7.20	−7.00	−6.60
**2NNA**	−7.40	−7.30	−7.60	−7.50	−7.10	−7.40	−7.20	−7.60	−7.90	−7.67
**2O72**	−6.60	−7.30	−8.20	−7.10	−6.90	−6.30	−6.30	−6.80	−6.50	−6.60
**2OC2**	−9.00	−8.80	−8.70	−8.90	−8.70	−9.20	−8.70	−9.00	−9.20	−8.63
**2OJ9**	−6.90	−7.70	−7.70	−6.90	−6.80	−7.00	−7.20	−7.70	−7.60	−7.23
**2OJI**	−7.10	−7.80	−8.60	−7.50	−8.00	−7.90	−7.50	−8.40	−8.10	−7.80
**2OJS**	−7.40	−7.10	−6.90	−6.90	−7.30	−6.70	−6.30	−7.00	−7.10	−6.97
**2OK5**	−8.30	−8.50	−8.90	−8.70	−8.20	−8.30	−8.30	−8.00	−8.50	−8.23
**2OKK**	−7.60	−7.40	−8.00	−7.40	−7.80	−7.50	−7.50	−7.70	−7.60	−7.37
**2OL2**	−7.90	−7.50	−7.70	−7.80	−7.60	−7.80	−7.50	−7.50	−8.00	−7.77
**2OVX**	−8.20	−8.50	−8.60	−8.20	−8.10	–	−8.20	−8.80	−8.40	−8.73
**2OW1**	−8.10	−8.40	−8.50	−8.20	−8.20	–	−8.40	−8.80	−8.50	−9.10
**2P33**	−7.80	−7.40	−7.80	−7.30	−7.50	−7.80	−7.00	−8.60	−8.20	−7.00
**2P3T**	−8.20	−7.70	−7.80	−8.10	−8.00	−7.90	−7.30	−7.50	−7.40	−7.40

**2P4E**	−8.40	−7.90	−9.40	−8.40	−8.40	−9.60	−7.60	−7.90	−8.90	−8.30
**2P4I**	−7.10	−7.00	−8.00	−7.00	−6.70	−7.40	−7.30	−7.00	−6.60	−6.80
**2P55**	−7.20	−6.70	−8.00	−7.40	−7.10	−8.50	−7.20	−7.20	−8.20	−7.03
**2PD5**	−7.40	−7.60	−7.30	−7.50	−7.20	−7.80	−7.10	−7.10	−7.70	−7.53
**2PIN**	−8.30	−8.50	−8.30	−8.40	−8.70	−8.40	−8.40	−8.50	−8.70	−8.50
**2PIV**	−7.40	−6.60	−7.30	−7.40	−6.80	−7.70	−6.90	−6.70	−7.20	−6.83
**2PIW**	−7.30	−7.90	−8.20	−7.30	−7.00	−7.60	−6.80	−7.00	−7.40	−7.30
**2PLL**	−7.60	−7.80	−8.60	−8.00	−8.60	−8.60	−7.40	−8.00	−8.50	−8.30
**2PPL**	−7.80	−7.60	−7.90	−7.40	−6.80	−8.20	−7.20	−7.10	−8.50	−7.00
**2PQ5**	−8.00	−7.80	−8.20	−8.10	−8.30	−7.80	−7.80	−7.60	−8.40	−8.10
**2PRG**	−8.10	−8.50	−8.60	−8.20	−7.80	–	−7.90	−8.40	−8.60	−8.67
**2PVR**	−7.70	−8.30	−8.60	−7.40	−7.10	–	−6.90	−9.20	−8.10	−6.80
**2Q8E**	−7.60	−7.50	−7.50	−7.70	−7.30	−7.60	−7.40	−8.20	−8.30	−7.33
**2QFO**	−7.20	−7.60	−7.40	−7.50	−7.50	−7.80	−7.90	−7.80	−7.20	−7.30
**2QG0**	−8.10	−8.10	−7.80	−8.30	−7.30	−7.80	−7.60	−8.70	−8.10	−7.30
**2QG2**	−8.00	−7.70	−7.90	−7.80	−7.40	−7.50	−7.30	−7.10	−7.50	−7.80
**2QKH**	−6.90	−7.40	−7.50	−7.00	−6.90	−7.60	−6.50	−7.10	−7.20	−6.97
**2QLD**	−6.30	−6.30	−6.50	−6.40	−6.60	−6.30	−5.90	−6.80	−6.50	−6.20
**2QMG**	−6.90	−7.20	−7.70	−6.80	−7.50	−7.70	−6.40	−8.20	−7.30	−7.13
**2QOE**	−7.90	−8.00	−8.00	−8.00	−7.70	−7.60	−7.80	−7.70	−7.80	−7.70
**2QON**	−7.50	−7.10	−7.50	−7.80	−7.20	–	−6.90	−7.20	−8.00	−6.93

**2QP8**	−6.90	−8.20	−8.40	−6.80	−6.90	−7.20	−6.60	−7.40	−7.50	−7.63
**2QRV**	−6.90	−6.90	−7.40	−6.90	−7.00	−7.40	−7.10	−7.70	−7.50	−7.43
**2QU5**	−6.80	−6.50	−7.00	−7.10	−7.20	−6.90	−6.60	−6.70	−6.90	−7.20
**2QW4**	−7.50	−7.60	−8.20	−7.60	−7.90	−8.00	−7.70	−8.00	−7.40	−7.63
**2R4B**	−6.80	−7.30	−7.30	−6.80	−6.40	–	−7.00	−6.70	−6.90	−6.67
**2RB4**	−6.80	−6.70	−6.80	−6.70	−7.30	−7.00	−7.60	−7.30	−7.10	−7.50
**2RH1**	−7.20	−7.90	−8.80	−7.90	−7.90	−7.50	−7.90	−7.80	−7.50	−8.87
**2RIP**	−8.00	−7.40	−7.80	−7.90	−7.20	−8.20	−7.50	−7.60	−7.90	−7.50
**2RIW**	−7.00	−7.30	−7.70	−7.00	−7.20	−7.80	−7.10	−7.00	−7.00	−7.13
**2RKU**	−8.80	−8.00	−8.60	−9.20	−7.60	–	−7.50	−8.40	−7.90	−7.50
**2ROX**	−6.80	−7.40	−7.40	−6.80	−6.70	−7.00	−6.60	−7.00	−7.10	−7.10
**2ROY**	−7.30	−7.20	−7.00	−7.10	−6.30	−6.70	−6.50	−7.00	−7.00	−6.97
**2UV5**	−8.10	−7.90	−7.50	−8.40	−7.50	−7.40	−7.10	−8.20	−8.20	−6.90
**2UW9**	−7.80	−8.60	−9.60	−7.60	−8.70	–	−7.90	−7.70	−8.70	−8.87
**2UWD**	−6.90	−6.70	−7.70	−6.80	−6.20	−6.80	−6.30	−6.50	−6.70	−6.80
**2V0M**	−8.20	−8.20	−9.30	−8.50	−7.90	−8.60	−9.00	−8.50	−8.70	−8.93
**2V0V**	−7.70	−7.40	−8.10	−7.70	−6.90	−8.00	−7.20	−7.10	−7.40	−7.47
**2V5Z**	−7.80	−7.40	−7.90	−8.10	−7.80	−7.80	−7.80	−7.80	−7.80	−7.50
**2V60**	−7.60	−7.70	−8.00	−7.90	−7.80	−8.00	−7.90	−7.60	−7.90	−7.60
**2V61**	−7.70	−7.50	−8.10	−8.00	−7.80	−8.10	−7.90	−7.80	−8.00	−7.60
**2V8Q**	−7.70	−7.70	−8.10	−8.30	−8.20	−8.30	−7.90	−8.00	−8.30	−8.40

**2VCJ**	−6.70	−6.30	−6.60	−6.70	−6.20	−6.80	−6.20	−6.50	−6.40	−6.33
**2VPF**	−8.10	−7.40	−7.80	−8.10	−7.50	−8.50	−7.80	−7.60	−7.50	−7.73
**2VQQ**	−8.70	−9.30	−9.40	−7.70	−8.20	−8.70	−8.60	−8.50	−8.70	−8.57
**2VRL**	−7.60	−8.10	−8.00	−7.90	−7.70	−7.80	−7.40	−7.60	−8.30	−7.47
**2VRM**	−7.50	−7.60	−8.10	−8.00	−7.40	−7.50	−7.60	−7.30	−8.00	−7.37
**2VWI**	−7.80	−7.60	−8.00	−8.10	−8.00	−9.00	−7.30	−7.70	−8.00	−7.73
**2VXT**	−7.60	−7.00	−7.20	−7.70	−7.20	−7.70	−7.20	−7.00	−7.20	−7.27
**2W1C**	−7.40	−8.80	−8.10	−7.70	−7.90	–	−7.40	−8.70	−8.80	−8.20
**2W26**	−8.40	−7.40	−7.50	−8.40	−7.00	−7.00	−6.80	−7.10	−7.00	−7.53
**2W3L**	−8.10	−8.50	−9.00	−8.40	−8.30	−8.40	−8.20	−8.40	−8.80	−7.53
**2W5B**	−8.50	−8.30	−8.20	−8.30	−8.60	–	−8.30	−8.50	−8.30	−7.70
**2W8Y**	−6.90	−8.30	−8.60	−7.20	−8.10	−8.60	−8.40	−7.30	−8.60	−8.47
**2WEW**	−6.40	−6.70	−6.90	−6.60	−7.00	−6.90	−6.60	−6.60	−7.00	−6.70
**2WEY**	−8.40	−8.60	−8.70	−8.40	−7.80	−7.50	−7.60	−8.50	−9.20	−7.80
**2WEZ**	−7.40	−7.30	−7.00	−7.20	−7.10	−7.40	−6.60	−7.50	−7.20	−6.87
**2WFX**	−8.20	−7.80	−8.60	−8.00	−8.60	−8.50	−8.40	−8.40	−9.30	−8.77
**2WGJ**	−7.50	−7.70	−8.10	−7.50	−7.20	–	−7.40	−8.80	−7.60	−7.33
**2WJO**	−8.40	−8.20	−8.20	−8.50	−7.50	−7.90	−7.40	−8.10	−8.10	−8.50
**2WKM**	−8.00	−8.60	−8.60	−8.60	−7.40	–	−8.00	−8.50	−8.90	−7.40
**2WRU**	−5.80	−6.00	−6.70	−5.90	−5.40	−5.70	−5.50	−6.30	−5.90	−6.00
**2WY3**	−8.20	−8.20	−8.30	−8.30	−8.50	−8.50	−8.50	−8.90	−8.60	−7.97

**2WZO**	−6.70	−6.40	−6.60	−6.50	−6.10	−6.80	−6.10	−6.10	−6.50	−5.90
**2X0G**	−8.10	−7.60	−7.80	−7.90	−7.50	−7.90	−7.40	−7.90	−8.10	−8.03
**2X0W**	−7.60	−8.00	−8.10	−7.80	−8.20	−8.00	−8.20	−8.10	−7.90	−8.40
**2X13**	−7.10	−7.10	−7.30	−7.00	−7.40	−7.30	−7.40	−7.00	−7.20	−7.90
**2X18**	−8.60	−8.40	−8.50	−8.80	−8.20	−8.10	−7.80	−8.70	−8.40	−8.50
**2X2L**	−6.80	−6.70	−7.50	−6.80	−6.80	−7.30	−6.80	−7.80	−6.80	−7.30
**2X39**	−9.40	−8.90	−9.30	−9.60	−9.20	−9.30	−9.50	−9.10	−9.50	−8.87
**2X4Z**	−7.90	−7.40	−8.20	−8.10	−6.60	−7.30	−7.20	−7.70	−7.10	−7.20
**2X57**	−7.30	−6.90	−7.90	−7.50	−7.30	−7.30	−7.20	−7.60	−7.30	−7.10
**2X70**	−8.40	−8.70	−8.70	−8.30	−9.10	−8.50	−8.70	−8.60	−9.30	−8.63
**2XA0**	−7.80	−7.70	−8.20	−7.70	−8.10	−8.10	−7.80	−7.50	−8.40	−8.23
**2XAC**	−7.10	−6.70	−7.40	−7.40	−6.70	−7.00	−6.60	−7.10	−7.50	−6.83
**2XIK**	−7.10	−7.20	−8.10	−6.70	−7.40	−8.00	−7.00	−8.00	−7.40	−6.53
**2XIR**	−8.30	−8.60	−9.10	−8.00	−8.10	−8.10	−8.30	−8.30	−8.60	−8.10
**2XK9**	−7.50	−7.10	−7.20	−7.00	−6.60	−7.10	−6.90	−6.90	−7.20	−7.13
**2XKW**	−7.80	−7.80	−8.00	−7.70	−7.50	−7.60	−7.50	−7.50	−7.90	−7.70
**2XN6**	−7.40	−7.00	−7.40	−6.90	−6.80	−7.60	−6.90	−7.20	−7.00	−6.90
**2XN7**	−7.40	−7.40	−8.40	−7.30	−7.20	−8.10	−8.70	−6.70	−7.90	−7.03
**2XN9**	−7.90	−7.50	−8.10	−7.70	−8.30	−8.50	−8.10	−8.10	−8.00	−8.00
**2XP2**	−7.30	−7.80	−7.30	−7.30	−6.90	–	−6.70	−7.60	−7.40	−6.87
**2XRA**	−7.80	−7.70	−7.60	−7.70	−7.60	−7.80	−7.60	−7.60	−8.10	−7.37

**2XRU**	−8.10	−8.40	−8.50	−8.40	−8.00	–	−7.40	−8.30	−8.80	−7.90
**2XSI**	−8.20	−7.70	−8.90	−8.90	−7.90	−8.50	−7.90	−8.10	−8.40	−8.10
**2XU7**	−8.60	−8.80	−9.20	−8.90	−9.00	−8.90	−8.80	−9.10	−9.50	−8.80
**2XUM**	−7.10	−7.20	−7.60	−7.20	−7.00	−7.30	−7.00	−7.40	−7.30	−6.80
**2XVT**	−8.30	−8.00	−8.80	−8.20	−8.00	−8.30	−7.80	−9.20	−8.60	−8.00
**2XYJ**	−8.20	−7.40	−8.40	−8.40	−7.50	−8.40	−7.40	−7.70	−7.70	−7.83
**2XYW**	−7.90	−8.40	−8.10	−7.90	−7.40	−8.10	−8.60	−7.10	−9.00	−9.23
**2XYX**	−7.20	−7.70	−8.30	−6.90	−7.80	−7.70	−8.00	−6.60	−7.90	−7.57
**2XZT**	−7.30	−7.50	−8.80	−8.80	−7.20	−8.00	−6.90	−7.40	−8.00	−7.33
**2Y1L**	−7.90	−7.70	−8.50	−8.00	−7.50	−8.00	−7.90	−7.70	−7.70	−7.40
**2Y7X**	−8.30	−7.70	−7.70	−8.40	−7.60	−7.50	−7.10	−7.40	−7.30	−7.80
**2YDO**	−7.40	−6.80	−7.60	−7.10	−7.00	−7.30	−6.70	−7.60	−7.10	−7.33
**2YDV**	−7.20	−7.40	−7.50	−6.70	−7.30	−7.30	−6.70	−7.40	−7.00	−6.90
**2YJA**	−7.00	−8.10	−8.50	−7.00	−6.60	−6.90	−6.80	−6.80	−6.90	−6.70
**2YJD**	−7.00	−7.00	−7.30	−7.30	−6.80	−7.20	−6.80	−6.80	−6.90	−7.13
**2YXJ**	−8.20	−7.50	−7.90	−8.00	−7.40	−7.60	−7.50	−7.40	−7.70	−7.87
**2Z5X**	−8.30	−7.60	−9.00	−8.10	−7.90	−8.10	−7.90	−7.30	−7.60	−7.53
**2Z5Y**	−7.60	−7.40	−7.70	−7.70	−7.30	−7.70	−8.70	−7.40	−7.40	−7.60
**2Z6W**	−7.10	−7.50	−7.30	−7.20	−8.10	−7.30	−6.90	−7.70	−7.80	−7.10
**2ZJW**	−7.50	−7.40	−8.10	−7.70	−7.40	−7.80	−7.50	−8.00	−7.50	−7.00
**2ZK1**	−7.20	−7.30	−7.80	−7.40	−6.80	–	−7.10	−7.10	−7.50	−6.93

**2ZZ0**	−8.40	−8.50	−9.10	−8.80	−8.80	−9.30	−8.80	−9.10	−8.70	−8.80
**3A5J**	−7.50	−7.50	−6.90	−7.80	−7.10	−7.10	−7.30	−7.40	−7.60	−7.17
**3A60**	−7.60	−7.30	−7.90	−7.40	−7.20	–	−7.40	−7.40	−7.70	−7.40
**3A62**	−6.70	−7.60	−8.50	−6.60	−7.20	−7.50	−7.30	−8.20	−7.50	−6.97
**3A8W**	−8.70	−8.50	−8.50	−8.40	−7.60	−8.10	−8.30	−8.20	−8.80	−8.23
**3AAA**	−7.50	−7.90	−8.20	−7.50	−7.40	−8.10	−7.40	−7.90	−8.00	−7.87
**3ADS**	−8.50	−7.70	−8.70	−7.70	−7.50	−8.00	−8.20	−7.60	−8.40	−8.50
**3ADT**	−7.60	−7.10	−7.90	−7.50	−7.50	−7.10	−7.70	−7.00	−7.80	−7.57
**3AJB**	−7.00	−6.80	−7.10	−6.80	−7.00	−6.80	−6.30	−6.80	−6.90	−7.10
**3AQQ**	−7.00	−7.10	−7.00	−6.90	−7.10	−6.90	−6.90	−7.50	−7.70	−6.83
**3AYU**	−7.00	−6.40	−6.20	−6.90	−6.60	−6.70	−6.70	−6.60	−6.60	−6.63
**3B3I**	−8.70	−9.00	−8.50	−8.70	−9.50	−8.60	−8.80	−8.70	−8.80	−8.53
**3B3K**	−7.20	−7.10	−7.80	−6.90	−7.40	−7.90	−7.40	−8.90	−8.40	−8.07
**3BCJ**	−7.70	−7.90	−7.60	−7.70	−7.10	−7.10	−6.70	−7.90	−8.10	−7.90
**3BEJ**	−8.00	−8.60	−8.30	−8.00	−7.70	−10.10	−8.00	−8.10	−8.20	−8.30
**3BG8**	−8.00	−7.90	−7.80	−8.20	−7.40	−7.40	−7.20	−8.20	−7.20	−7.37
**3BM9**	−6.80	−6.20	−6.80	−6.70	−6.30	−7.30	−6.20	−6.40	−6.70	−6.53
**3BMY**	−8.00	−7.50	−8.10	−8.10	−6.90	−7.30	−6.90	−7.70	−7.90	−6.70
**3BQD**	−7.30	−7.30	−7.10	−7.40	−7.90	−7.10	−7.40	−6.80	−6.90	−7.30
**3BQR**	−7.50	−7.60	−7.30	−7.40	−7.80	−7.90	−7.40	−7.70	−7.70	−7.60
**3BRT**	−6.60	−6.30	−6.20	−6.40	−6.30	−6.30	−7.30	−6.50	−6.20	−6.17

**3BRV**	−6.60	−6.60	−6.70	−7.10	−6.40	−6.30	−6.30	−6.80	−6.70	−6.97
**3BTR**	−7.90	−7.20	−7.60	−7.80	−7.20	−7.20	−7.10	−7.40	−7.90	−7.10
**3BWM**	−6.40	−6.00	−6.10	−6.40	−6.00	−6.20	−6.00	−6.00	−6.00	−6.53
**3C0Z**	−8.50	−8.00	−9.10	−8.40	−8.20	−8.10	−8.30	−8.30	−8.60	−7.83
**3C10**	−8.60	−8.30	−9.20	−8.80	−8.30	−8.40	−8.30	−8.40	−8.70	−7.70
**3C4C**	−7.60	−8.80	−9.10	−7.70	−7.60	−8.20	−7.80	−9.20	−8.80	−7.97
**3CDP**	−7.40	−8.30	−8.10	−7.30	−7.40	−9.00	−7.10	−8.00	−8.30	−7.50
**3CDS**	−7.10	−6.70	−8.60	−7.30	−6.90	−8.40	−8.10	−7.70	−7.60	−7.23
**3CJW**	−7.10	−6.80	−6.70	−6.70	−6.80	−6.50	−6.30	−7.10	−6.50	−6.40
**3CO6**	−7.90	−8.00	−8.30	−8.60	−8.60	−8.40	−8.20	−8.80	−8.80	−7.97
**3CQU**	−7.00	−7.00	−7.70	−7.00	−6.70	−6.80	−6.30	−7.10	−7.00	−6.77
**3D24**	−8.50	−8.60	−8.40	−8.00	−8.00	−8.20	−7.80	−8.90	−8.60	−8.43
**3D6D**	−7.50	−8.00	−8.30	−7.40	−8.00	−8.90	−8.10	−9.00	−7.60	−8.20
**3D90**	−8.20	−7.90	−8.60	−8.00	−7.90	−9.20	−8.80	−7.10	−9.00	−8.13
**3D9I**	−7.40	−8.20	−8.70	−7.60	−7.50	−7.60	−7.10	−8.50	−8.40	−7.50
**3DEH**	−8.20	−8.00	−8.60	−8.60	−7.70	−8.30	−7.40	−8.10	−8.00	−7.47
**3DY7**	−7.10	−8.40	−7.80	−7.50	−7.90	−8.10	−7.60	−7.90	−8.60	−7.50
**3E7G**	−7.80	−8.00	−10.10	−7.90	−7.60	–	−7.50	−8.70	−8.20	−8.13
**3E8N**	−8.30	−8.20	−8.20	−7.90	−8.50	–	−8.00	−8.40	−8.40	−8.13
**3EAH**	−8.40	−8.70	−10.70	−9.10	−8.40	−8.20	−8.70	−9.60	−8.70	−9.07
**3EAX**	−7.90	−7.80	−8.10	−7.70	−7.70	−7.50	−7.20	−8.50	−8.20	−7.57

**3ECS**	−8.40	−8.00	−8.60	−7.80	−8.00	−7.80	−7.80	−8.10	−8.30	−8.33
**3EKO**	−8.00	−8.00	−8.50	−8.20	−7.60	−7.60	−7.60	−7.40	−8.20	−7.20
**3EKR**	−7.90	−7.60	−7.60	−7.90	−7.70	−8.00	−7.70	−7.30	−8.10	−7.30
**3EML**	−7.40	−7.40	−7.70	−7.60	−6.60	−7.30	−7.30	−8.00	−7.90	−6.97
**3EO2**	−6.30	−6.70	−7.00	−6.40	−6.70	−6.80	−6.30	−6.30	−7.20	−6.40
**3EO9**	−7.40	−7.60	−7.60	−7.50	−7.40	−7.30	−7.60	−7.30	−7.40	−7.73
**3EOX**	−6.70	−6.60	−6.90	−6.80	−6.50	−7.60	−6.30	−6.90	−6.60	−6.87
**3EQB**	−7.30	−6.90	−7.90	−7.30	−6.80	–	−7.00	−7.40	−8.00	−7.00
**3EQC**	−8.70	−7.90	−8.10	−8.30	−8.30	–	−7.40	−8.10	−7.90	−8.70
**3EQH**	−8.80	−8.30	−8.40	−8.70	−8.60	–	−8.40	−8.50	−8.50	−8.10
**3EQM**	−8.30	−8.10	−8.40	−8.40	−8.50	−9.40	−7.80	−7.60	−8.50	−7.90
**3ERT**	−7.50	−6.80	−7.30	−7.30	−7.30	−7.40	−7.30	−7.40	−7.60	−7.60
**3ET3**	−7.70	−8.30	−7.30	−7.50	−7.00	−7.10	−7.00	−8.70	−8.60	−7.13
**3EW8**	−7.10	−6.90	−7.10	−7.00	−6.80	−6.80	−6.40	−7.00	−6.80	−6.60
**3EWV**	−6.70	−6.60	−6.70	−7.10	−6.30	−6.90	−6.50	−6.60	−6.80	−6.20
**3EYG**	−6.90	−7.10	−7.70	−6.70	−6.40	−7.00	−7.10	−7.10	−7.10	−6.73
**3F07**	−7.20	−8.10	−7.70	−7.50	−7.60	−8.90	−8.30	−7.40	−7.20	−7.90
**3F17**	−6.70	−7.40	−7.30	−7.00	−7.50	–	−7.30	−7.50	−7.50	−8.13
**3F6K**	−7.40	−7.20	−7.80	−7.60	−7.30	−7.30	−7.20	−7.50	−8.30	−7.30
**3F6Q**	−6.60	−6.70	−6.60	−6.60	−6.50	−6.90	−6.60	−6.70	−6.80	−7.20
**3F88**	−7.60	−8.40	−8.40	−8.00	−7.60	−8.80	−7.60	−9.00	−8.10	−7.47

**3F8S**	−8.30	−7.80	−7.80	−8.40	−7.30	−8.60	−7.60	−7.90	−7.80	−7.63
**3FAY**	−7.10	−6.90	−7.60	−7.10	−6.70	−7.30	−7.10	−7.80	−6.80	−6.63
**3FF8**	−7.50	−7.20	−7.80	−7.30	−7.10	–	−7.40	−7.40	−7.40	−7.60
**3FGU**	−7.30	−7.00	−8.40	−7.50	−8.00	–	−7.90	−8.10	−8.10	−7.73
**3FQX**	−8.20	−9.30	−8.70	−9.00	−8.70	−8.40	−8.70	−8.20	−9.00	−8.30
**3FR0**	−8.00	−7.70	−7.80	−7.60	−8.00	−7.90	−8.00	−7.40	−8.10	−7.80
**3FT8**	−8.30	−8.70	−9.50	−8.80	−7.00	−7.50	−7.40	−7.70	−9.20	−7.80
**3G04**	−7.40	−7.60	−7.50	−7.40	−7.30	−7.60	−6.90	−7.50	−7.90	−7.23
**3G0E**	−8.00	−7.40	−8.60	−8.00	−8.00	−8.70	−7.60	−8.30	−8.40	−7.77
**3G42**	−7.30	−7.50	−8.20	−7.30	−7.40	–	−8.00	−8.00	−7.50	−7.80
**3G9E**	−7.90	−7.80	−7.90	−7.70	−7.00	−7.80	−7.60	−7.70	−8.00	−7.80
**3GCW**	−7.30	−6.80	−7.20	−7.10	−7.80	−7.20	−7.50	−7.20	−8.70	−7.37
**3GGF**	−7.50	−7.60	−8.10	−7.60	−7.30	−7.20	−7.30	−8.50	−8.10	−7.23
**3GOI**	−8.50	−7.90	−8.20	−8.70	−8.20	−7.90	−7.70	−8.00	−8.40	−7.40
**3GWS**	−7.60	−6.90	−7.50	−7.80	−7.10	−7.70	−7.40	−7.70	−7.70	−7.30
**3GWX**	−7.40	−7.80	−8.10	−7.80	−7.00	−7.90	−7.90	−7.90	−7.70	−7.77
**3GZ9**	−8.10	−7.80	−8.00	−8.40	−7.60	−8.00	−7.00	−7.50	−7.50	−7.13
**3H1V**	−7.70	−8.50	−8.00	−7.80	−7.70	−7.60	−7.30	−7.90	−8.90	−9.17
**3HDN**	−7.10	−7.70	−8.10	−7.60	−7.80	−9.10	−6.80	−8.80	−8.30	−7.43
**3HGZ**	−8.00	−7.70	−8.70	−8.00	−7.60	−8.20	−8.40	−8.00	−7.90	0.00
**3HHU**	−7.90	−7.80	−7.90	−8.00	−7.40	−7.70	−7.40	−7.80	−7.80	−7.83

**3HNG**	−7.60	−7.20	−7.40	−7.90	−8.20	−8.00	−8.20	−7.50	−7.80	−7.73
**3HOD**	−7.60	−7.50	−8.10	−7.70	−7.70	−8.10	−7.70	−7.70	−8.30	−7.37
**3HOK**	−8.20	−7.90	−8.70	−8.50	−8.30	−8.00	−7.50	−7.90	−8.30	−8.70
**3HXG**	−7.90	−8.00	−7.60	−7.80	−7.80	−8.30	−7.70	−8.20	−8.40	−7.30
**3IAI**	−9.20	−8.80	−8.90	−9.40	−9.10	−8.80	−8.40	−9.20	−9.10	−8.70
**3IEO**	−7.00	−7.10	−7.20	−7.20	−7.00	−7.70	−6.80	−6.90	−7.00	−7.30
**3IIT**	−8.30	−8.10	−8.00	−8.20	−7.60	−7.40	−7.00	−7.80	−7.10	−8.43
**3IJE**	−9.00	−7.80	−8.70	−8.90	−7.90	−8.90	−8.00	−8.00	−7.80	−8.33
**3ILZ**	−8.40	−7.50	−8.20	−8.30	−6.90	−8.20	−7.40	−7.70	−8.30	−7.30
**3IMX**	−7.80	−7.90	−8.90	−8.00	−8.00	−8.60	−8.80	−8.90	−8.50	−7.60
**3INH**	−8.50	−7.90	−8.10	−8.90	−7.90	−7.90	−8.00	−7.60	−8.10	−8.37
**3IOL**	−6.90	−7.10	−7.10	−6.60	−6.30	−6.90	−7.20	−6.90	−7.20	−6.87
**3IPB**	−6.90	−6.70	−7.20	−7.20	−6.50	−7.00	−6.40	−7.60	−6.80	−6.37
**3IPS**	−8.40	−8.20	−9.00	−8.10	−7.70	−8.00	−7.60	−8.10	−8.00	−7.80
**3J0A**	−7.30	−7.20	−8.00	−7.30	−8.60	–	−7.70	−7.30	−7.20	−7.53
**3JRW**	−7.50	−7.50	−8.00	−7.30	−7.40	−7.30	−7.40	−7.60	−7.80	−7.67
**3JS2**	−7.90	−7.90	−8.50	−8.00	−7.90	−8.50	−7.60	−8.40	−8.40	−7.90
**3JUI**	−7.10	−7.90	−8.40	−7.00	−6.60	−7.60	−7.30	−7.80	−8.20	−7.20
**3JXU**	−7.40	−7.30	−8.10	−7.50	−8.00	−8.20	−7.60	−8.70	−7.90	−7.90
**3JZC**	−7.50	−7.70	−7.50	−7.30	−7.20	−7.40	−6.90	−7.20	−7.40	−7.20
**3K6P**	−7.40	−6.60	−6.70	−7.30	−6.70	−6.70	−6.20	−7.00	−6.70	−6.70

**3K97**	−7.80	−7.70	−7.30	−7.80	−7.10	−7.30	−7.30	−7.50	−7.20	−6.93
**3KCG**	−8.00	−7.80	−8.50	−8.10	−7.90	−8.10	−7.70	−8.30	−8.30	−7.87
**3KEK**	−6.90	−7.30	−7.60	−7.20	−7.80	−6.90	−6.70	−6.90	−7.80	−7.17
**3KIJ**	−7.30	−7.60	−8.00	−7.40	−7.10	−7.60	−8.00	−7.60	−7.80	−7.20
**3KMC**	−7.50	−7.10	−7.80	−7.40	−7.40	–	−7.50	−8.20	−7.30	−8.07
**3KPJ**	−7.80	−7.40	−8.00	−7.80	−7.20	−7.40	−6.90	−6.80	−7.50	−6.63
**3KQR**	−7.10	−7.10	−7.50	−6.70	−7.20	−7.20	−7.40	−7.60	−7.30	−7.33
**3KRW**	−7.70	−7.20	−7.70	−8.00	−8.10	−9.00	−8.20	−8.10	−8.00	−8.00
**3KVW**	−7.90	−8.50	−9.70	−8.00	−7.60	−8.00	−7.40	−9.30	−8.50	−7.10
**3KWA**	−7.30	−6.70	−7.40	−7.30	−6.70	−7.00	−6.70	−6.80	−7.10	−7.30
**3KYT**	−8.50	−8.10	−9.10	−8.00	−7.70	−9.70	−9.20	−7.70	−8.30	−8.30
**3L0J**	−8.60	−8.10	−8.50	−8.40	−9.00	−9.00	−8.50	−7.70	−8.80	−9.10
**3L0L**	−9.00	−8.80	−9.10	−8.70	−9.70	−9.00	−9.00	−9.00	−9.00	−9.10
**3L0V**	−7.40	−7.30	−7.80	−7.50	−7.40	–	−7.90	−8.00	−7.30	−7.60
**3L2Y**	−7.30	−7.20	−7.90	−7.90	−7.80	−8.20	−7.40	−7.30	−7.60	−7.50
**3L3X**	−7.20	−7.10	−7.20	−7.20	−7.00	−7.50	−6.70	−7.30	−7.40	−7.37
**3L4W**	−7.70	−7.60	−8.10	−7.70	−8.00	−7.40	−7.50	−8.10	−7.80	−7.80
**3L54**	−8.40	−8.30	−8.80	−8.50	−8.60	−8.80	−8.40	−8.50	−9.20	−8.23
**3L9J**	−7.70	−8.10	−8.00	−7.30	−7.10	−7.60	−7.20	−8.20	−7.80	−7.40
**3L9N**	−7.50	−7.70	−7.80	−7.50	−6.90	−7.80	−6.90	−9.10	−8.20	−7.07
**3LBD**	−6.90	−7.20	−7.50	−7.30	−7.70	−6.90	−6.80	−7.80	−6.60	−7.30

**3LBO**	−7.70	−7.80	−9.00	−7.90	−7.60	−7.40	−7.00	−7.40	−7.90	−7.70
**3LDQ**	−8.00	−9.00	−9.40	−9.00	−8.70	−9.00	−8.50	−9.30	−8.70	−8.50
**3LDX**	−8.70	−7.50	−8.20	−8.40	−8.00	−7.50	−7.10	−7.20	−8.20	−7.67
**3LE9**	−7.40	−7.50	−7.50	−7.30	−7.20	–	−8.20	−8.50	−7.40	−7.73
**3LEV**	−7.40	−7.20	−7.70	−7.40	−7.50	−7.50	−7.50	−7.50	−7.30	−7.30
**3LKJ**	−8.00	−8.40	−9.10	−7.90	−8.30	−9.00	−7.70	−8.60	−8.30	−8.63
**3LQ8**	−8.10	−8.20	−8.00	−8.20	−7.50	–	−7.40	−7.90	−8.10	−7.90
**3LQM**	−7.10	−6.80	−7.60	−7.60	−6.90	−7.00	−7.30	−6.80	−7.30	−7.10
**3LV3**	−7.90	−8.10	−8.30	−8.40	−8.30	−9.00	−8.80	−7.90	−8.60	−8.37
**3LZB**	−7.30	−8.10	−7.80	−8.00	−7.20	–	−7.20	−7.50	−7.40	−7.73
**3MAP**	−7.70	−8.10	−8.30	−8.10	−8.00	−7.70	−8.40	−7.80	−8.50	−7.50
**3MAX**	−8.10	−7.30	−8.70	−8.20	−8.10	−8.40	−8.30	−8.00	−8.50	−8.00
**3MBL**	−7.50	−7.70	−8.40	−7.70	−6.90	–	−7.20	−8.50	−8.00	−8.73
**3MDY**	−7.60	−8.00	−8.20	−7.80	−7.70	−7.90	−7.90	−8.30	−8.00	−7.53
**3MJ1**	−6.60	−6.50	−7.10	−6.70	−7.00	−6.90	−6.40	−7.50	−6.80	−6.77
**3MJG**	−7.00	−6.80	−7.30	−6.90	−6.80	−6.90	−6.70	−6.90	−7.10	−6.70
**3MK8**	−7.10	−6.80	−6.90	−7.10	−6.50	−6.30	−6.90	−6.80	−6.80	−7.50
**3MUP**	−8.10	−8.20	−8.40	−7.80	−7.70	−8.20	−7.60	−8.30	−7.90	−7.30
**3MVH**	−8.90	−8.70	−8.90	−8.60	−8.70	–	−8.70	−9.30	−8.70	−8.40
**3MWD**	−7.90	−8.60	−8.50	−7.90	−8.00	−7.80	−7.90	−8.40	−7.90	−8.30
**3N3F**	−6.40	−6.50	−6.30	−6.50	−5.60	−6.00	−5.70	−6.40	−6.10	−5.90

**3NBV**	−8.40	−7.80	−8.90	−8.40	−8.20	−8.70	−8.20	−8.30	−9.20	−8.03
**3NG8**	−6.70	−6.40	−6.20	−6.30	−6.60	−6.20	−6.20	−6.10	−6.60	−6.10
**3NL7**	−7.00	−6.40	−7.30	−6.90	−6.30	−7.20	−6.50	−6.70	−7.00	−7.23
**3NMM**	−8.50	−8.40	−8.10	−8.20	−8.20	−8.10	−8.00	−8.20	−8.80	−8.00
**3NMQ**	−8.70	−9.20	−9.10	−9.00	−7.90	−8.20	−7.20	−7.90	−9.60	−7.57
**3NPC**	−7.20	−7.20	−7.40	−7.20	−7.40	−7.30	−7.40	−8.20	−7.60	−7.87
**3NXP**	−7.80	−7.40	−7.70	−7.70	−7.20	−7.90	−7.50	−7.50	−7.60	−7.50
**3NXQ**	−9.10	−9.00	−9.00	−8.90	−8.80	−8.80	−8.70	−9.00	−8.90	−8.67
**3O0I**	−9.00	−9.50	−9.30	−9.00	−7.80	−8.20	−7.50	−7.90	−9.70	−7.77
**3O3U**	−10.10	−8.50	−10.20	−9.50	−8.60	−9.30	−9.00	−10.00	−9.10	−8.77
**3O5X**	−7.40	−6.50	−7.30	−7.50	−7.20	−6.90	−7.10	−7.10	−6.70	−7.10
**3O96**	−7.90	−9.10	−9.30	−8.00	−7.80	−8.50	−8.50	−8.40	−9.70	−8.77
**3OB7**	−8.40	−8.00	−8.90	−8.50	−8.50	−8.20	−8.20	−8.10	−8.20	−8.40
**3OCB**	−8.30	−8.60	−8.70	−8.30	−8.50	−8.50	−8.40	−8.80	−8.80	−8.27
**3OD5**	−6.90	−7.40	−7.80	−7.00	−6.60	−7.60	−6.90	−7.20	−7.40	−7.10
**3ODW**	−7.20	−7.60	−7.50	−7.10	−7.10	−8.00	−7.70	−7.70	−7.30	−7.00
**3OFS**	−7.90	−8.60	−8.60	−8.50	−8.20	−8.40	−8.10	−8.70	−8.40	−8.27
**3OJ3**	−8.40	−8.20	−8.90	−8.60	−8.70	−8.90	−8.30	−8.90	−8.80	−8.60
**3OLD**	−8.90	−9.00	−8.90	−8.90	−8.60	−9.00	−9.30	−8.60	−9.20	−8.20
**3OLL**	−8.20	−8.10	−8.20	−8.30	−7.70	−8.30	−7.80	−8.00	−7.70	−8.00
**3OLS**	−7.40	−7.80	−8.40	−8.20	−7.70	−8.00	−8.10	−7.80	−7.60	−7.47

**3OQ5**	−8.70	−8.30	−8.20	−8.00	−8.40	−8.20	−8.20	−8.40	−8.70	−8.80
**3OS3**	−7.60	−9.10	−8.10	−7.40	−7.20	−7.70	−6.80	−8.20	−7.60	−7.33
**3OW4**	−8.30	−8.40	−8.70	−8.00	−8.50	−8.20	−7.90	−8.70	−8.50	−8.23
**3OWD**	−7.80	−7.40	−7.60	−7.70	−6.80	–	−7.00	−7.10	−7.10	−8.13
**3OY8**	−7.30	−7.40	−7.10	−6.80	−6.50	−6.60	−7.00	−7.30	−7.00	−6.87
**3OZK**	−6.80	−6.40	−7.00	−7.00	−6.50	−6.40	−6.30	−7.10	−6.90	−6.50
**3P0U**	−7.40	−7.60	−8.60	−7.40	−6.90	−7.30	−6.80	−7.70	−7.10	−8.03
**3P1D**	−8.00	−7.20	−7.90	−7.60	−6.70	−7.40	−6.80	−7.50	−7.40	−7.50
**3PL6**	−7.30	−7.50	−8.20	−7.60	−7.30	−7.40	−7.80	−7.50	−7.70	−7.63
**3PLZ**	−9.30	−8.80	−9.40	−9.30	−9.40	−9.50	−8.60	−8.30	−8.70	−8.70
**3PM0**	−8.20	−7.90	−8.20	−8.60	−8.20	−7.90	−7.40	−8.00	−7.80	−7.80
**3PP0**	−7.30	−8.10	−8.00	−7.70	−7.60	−7.30	−7.40	−8.90	−8.30	−7.60
**3PP1**	−7.80	−7.20	−8.60	−8.40	−8.60	–	−8.20	−8.00	−8.60	−8.40
**3PSL**	−8.60	−8.20	−8.70	−8.20	−8.00	−8.20	−8.60	−8.80	−8.80	−8.80
**3PTA**	−7.10	−7.30	−7.90	−7.50	−7.90	−8.30	−7.90	−7.70	−7.90	−8.03
**3PTA**	−7.10	−7.30	−7.90	−7.50	−7.90	−8.30	−7.90	−7.70	−7.90	−8.03
**3PUF**	−7.60	−7.70	−8.70	−8.00	−7.90	−8.30	−8.00	−8.20	−8.00	−7.97
**3Q93**	−7.30	−8.40	−8.30	−7.40	−7.20	−7.70	−9.00	−7.30	−7.60	−8.23
**3QAK**	−7.30	−7.00	−7.70	−7.40	−7.20	−7.20	−6.70	−7.70	−7.50	−6.80
**3QBJ**	−8.00	−7.60	−7.80	−8.10	−7.50	−8.10	−8.00	−7.90	−7.60	−8.10
**3QD6**	−8.20	−7.40	−8.30	−8.20	−7.20	−7.60	−7.50	−7.50	−7.40	−7.60

**3QKL**	−8.60	−8.90	−9.00	−8.90	−9.80	−9.30	−8.70	−9.00	−8.90	−9.00
**3QM4**	−8.60	−7.80	−8.60	−9.00	−8.50	−8.60	−8.10	−8.50	−8.30	−8.23
**3QOA**	−7.80	−6.80	−7.80	−7.40	−6.90	−7.50	−6.70	−7.00	−7.50	−7.50
**3QU8**	−7.80	−7.20	−8.60	−8.60	−7.70	−7.80	−7.50	−7.40	−7.60	−8.17
**3QWC**	−7.80	−7.50	−8.60	−7.80	−7.60	−7.50	−6.80	−7.30	−7.70	−7.17
**3QX3**	−7.90	−7.80	−9.00	−8.60	−8.80	−9.10	−8.70	−8.90	−7.60	−9.07
**3RQD**	−7.10	−7.00	−7.70	−7.40	−7.20	−7.50	−7.20	−6.70	−6.90	−7.17
**3RUK**	−8.80	−8.00	−8.30	−8.70	−8.10	−9.10	−7.70	−7.80	−7.90	−8.30
**3S95**	−8.20	−8.80	−9.00	−8.40	−8.20	−8.90	−7.80	−8.70	−9.20	−8.07
**3SOR**	−7.80	−7.10	−7.50	−7.90	−7.20	−7.50	−7.50	−7.40	−7.50	−7.10
**3SOS**	−7.60	−7.70	−8.00	−7.80	−7.40	−7.40	−7.40	−7.30	−7.60	−7.10
**3SW2**	−8.50	−8.00	−8.00	−8.50	−7.70	−7.50	−6.90	−7.40	−7.50	−8.10
**3SWR**	−7.40	−7.50	−8.20	−8.10	−7.90	−8.10	−7.90	−7.90	−8.00	−8.90
**3T03**	−8.30	−8.00	−8.50	−8.30	−8.30	−9.20	−8.30	−8.30	−8.40	−8.27
**3TH5**	−7.80	−7.80	−7.90	−7.30	−7.50	−7.60	−7.40	−7.60	−7.40	−7.80
**3TKM**	−8.00	−7.50	−8.10	−8.60	−7.90	–	−7.90	−7.40	−8.10	−7.80
**3TOP**	−7.40	−6.60	−7.30	−7.00	−7.30	−7.40	−8.20	−7.40	−6.80	−7.10
**3TXO**	−7.80	−9.20	−9.50	−7.80	−7.70	−8.50	−8.30	−9.00	−8.50	−7.93
**3TY0**	−8.10	−7.80	−7.80	−8.20	−7.60	−7.80	−7.50	−8.60	−8.40	−7.60
**3U4S**	−7.80	−7.60	−7.70	−7.60	−7.70	−7.80	−7.80	−8.00	−7.70	−7.43
**3U6I**	−7.30	−7.40	−6.70	−7.10	−6.50	–	−6.50	−6.90	−7.70	−7.07

**3U9Q**	−7.40	−7.20	−7.40	−7.10	−7.00	−6.60	−6.60	−8.10	−7.90	−7.00
**3UA1**	−9.20	−9.60	−9.00	−9.00	−8.40	−9.50	−8.20	−8.70	−9.80	−8.60
**3UA5**	−8.70	−9.10	−8.80	−9.00	−7.70	−9.00	−7.90	−9.10	−8.20	−8.37
**3UEE**	−7.10	−6.50	−7.10	−6.60	−6.40	−6.70	−6.50	−6.80	−7.10	−6.50
**3UIH**	−6.60	−6.80	−6.90	−6.80	−6.50	−6.90	−6.70	−6.80	−7.00	−6.80
**3UIU**	−7.60	−7.50	−8.30	−8.00	−7.40	−7.50	−7.70	−7.80	−7.90	−7.30
**3UL7**	−6.40	−6.90	−6.30	−6.50	−6.60	–	−6.40	−6.70	−6.80	−7.00
**3UL8**	−6.70	−7.20	−7.00	−7.00	−7.20	–	−6.90	−6.90	−7.00	−7.67
**3UL9**	−6.90	−6.80	−6.70	−7.10	−6.30	–	−7.00	−7.30	−6.70	−6.30
**3ULA**	−7.70	−8.30	−8.70	−8.30	−7.80	–	−8.50	−7.70	−8.50	−8.40
**3UNJ**	−7.80	−7.00	−7.50	−8.10	−7.00	−6.90	−7.20	−7.90	−7.30	−7.17
**3UO5**	−8.50	−8.70	−8.00	−8.70	−7.50	−8.00	−7.50	−8.40	−8.80	−7.30
**3UOK**	−8.90	−8.10	−8.60	−8.50	−8.40	−8.20	−7.80	−7.90	−8.20	−8.37
**3UQP**	−6.70	−6.50	−6.80	−6.90	−7.30	−6.90	−6.70	−6.90	−6.80	−6.73
**3UT3**	−8.70	−9.00	−9.40	−8.70	−9.00	−9.10	−9.20	−8.90	−9.20	−8.70
**3UVV**	−7.80	−8.00	−8.20	−7.80	−7.90	−8.20	−7.70	−8.00	−8.00	−7.70
**3UYT**	−7.40	−7.50	−8.20	−7.90	−7.60	−8.20	−7.50	−8.30	−7.80	−7.80
**3V6R**	−7.40	−7.70	−8.30	−7.30	−7.60	−7.80	−7.80	−8.90	−8.50	−7.60
**3VEV**	−8.50	−8.00	−8.00	−8.30	−7.90	−8.60	−7.90	−8.40	−8.10	−7.67
**3VEY**	−8.60	−8.40	−7.90	−8.40	−8.00	−8.30	−8.30	−8.00	−8.10	−7.60
**3VHE**	−7.90	−8.10	−9.00	−7.60	−8.30	−8.70	−8.50	−7.70	−8.40	−8.20

**3VI8**	−8.10	−8.40	−7.90	−8.10	−6.60	−10.10	−6.50	−7.70	−7.30	−6.37
**3VJH**	−8.00	−7.70	−7.90	−7.80	−7.20	−7.50	−7.50	−7.60	−8.10	−7.20
**3VJI**	−7.60	−8.00	−7.90	−7.60	−7.40	−8.40	−7.60	−7.60	−8.00	−7.37
**3VKX**	−7.20	−6.40	−6.90	−6.80	−6.40	−6.70	−6.30	−6.90	−6.80	−6.23
**3VVH**	−7.90	−7.90	−8.30	−8.30	−8.30	–	−7.80	−8.70	−8.50	−7.80
**3VW6**	−7.10	−7.20	−7.30	−7.00	−7.20	−7.00	−6.40	−7.40	−8.00	−6.70
**3VW7**	−10.10	−7.80	−8.70	−8.10	−9.20	–	−7.70	−8.30	−9.00	−8.73
**3W33**	−7.70	−8.30	−8.30	−8.20	−7.30	−7.10	−7.20	−9.00	−8.50	−7.30
**3W3G**	−7.80	−7.90	−8.00	−8.40	−8.00	–	−8.70	−8.70	−8.00	−8.10
**3W3J**	−8.60	−8.70	−8.40	−8.60	−8.60	–	−8.50	−9.90	−9.10	−8.57
**3W3K**	−8.80	−8.50	−9.30	−8.40	−8.20	–	−8.50	−8.70	−8.40	−8.23
**3W3L**	−8.00	−7.70	−8.20	−8.30	−7.20	–	−7.50	−7.90	−8.00	−8.27
**3W3M**	−8.30	−8.30	−8.90	−8.50	−7.70	–	−7.90	−8.30	−8.30	−8.53
**3W3N**	−9.20	−8.50	−8.80	−9.00	−8.40	–	−8.40	−9.00	−8.70	−8.70
**3WHW**	−7.30	−8.60	−8.80	−7.60	−7.80	−8.60	−7.20	−7.90	−8.50	−9.13
**3WN4**	−8.20	−8.10	−8.70	−8.40	−8.10	–	−7.30	−7.90	−8.70	−8.63
**3WQH**	−7.90	−7.70	−8.00	−7.80	−7.50	−8.10	−8.00	−7.50	−7.70	−7.50
**3X36**	−7.90	−9.10	−8.90	−8.50	−7.70	−8.30	−9.90	−7.00	−8.80	−8.80
**3ZBF**	−7.20	−7.60	−7.40	−7.30	−6.60	−7.00	−6.90	−7.90	−7.60	−7.00
**3ZEP**	−7.30	−7.20	−8.60	−7.20	−7.90	−8.50	−7.60	−7.80	−7.90	−7.83
**3ZGV**	−8.50	−7.80	−8.90	−8.70	−7.70	−8.10	−8.00	−8.00	−8.00	−8.13

**3ZLN**	−7.00	−7.10	−7.60	−7.30	−6.50	−7.00	−6.30	−6.90	−7.40	−6.73
**3ZME**	−7.50	−7.90	−7.80	−7.50	−7.90	–	−7.60	−8.00	−7.70	−8.00
**3ZNS**	−8.20	−8.90	−8.50	−8.40	−8.30	−8.10	−8.20	−8.70	−8.80	−7.73
**3ZR0**	−7.30	−8.90	−8.00	−7.90	−7.30	−7.40	−7.40	−7.50	−8.10	−8.00
**3ZR1**	−7.60	−8.70	−8.00	−7.50	−7.30	−7.50	−7.40	−7.50	−8.10	−7.83
**3ZXZ**	−8.90	−7.60	−8.00	−8.60	−7.40	–	−6.80	−7.50	−7.20	−7.63
**3ZZE**	−8.70	−7.70	−7.90	−8.30	−6.80	–	−7.50	−8.20	−7.50	−7.83
**4A5S**	−8.10	−7.70	−8.00	−8.20	−7.70	−7.90	−7.60	−7.70	−8.10	−8.07
**4A7A**	−7.70	−7.90	−8.00	−7.80	−7.80	−8.00	−7.80	−7.60	−8.00	−7.80
**4A7I**	−7.40	−8.30	−7.40	−7.30	−6.80	−7.10	−7.30	−8.00	−7.20	−6.93
**4ACG**	−8.00	−8.70	−8.20	−8.50	−8.10	−8.60	−8.00	−9.00	−8.00	−8.17
**4AG8**	−8.30	−8.40	−9.10	−7.90	−8.00	−8.10	−8.20	−8.30	−8.70	−8.03
**4AJU**	−8.20	−8.30	−8.60	−8.10	−7.40	−7.50	−7.40	−7.60	−8.10	−7.30
**4AL0**	−6.30	−6.40	−6.40	−6.20	−6.00	–	−6.20	−6.10	−6.50	−6.20
**4AOJ**	−8.10	−8.90	−11.00	−8.10	−7.70	−8.00	−8.10	−10.00	−9.30	−7.97
**4ASD**	−8.10	−7.80	−8.40	−7.80	−8.40	−8.10	−7.90	−7.90	−8.00	−7.87
**4ASK**	−7.60	−7.30	−7.80	−7.90	−7.80	−7.50	−7.40	−8.20	−8.50	−7.50
**4ASX**	−8.20	−7.60	−8.40	−7.80	−7.80	−8.30	−8.60	−8.10	−7.60	−7.83
**4B0O**	−9.80	−9.60	−9.60	−9.80	−9.10	−9.00	−8.80	−9.10	−9.60	−9.40
**4B0Q**	−8.60	−8.60	−8.50	−8.90	−8.40	−9.20	−8.50	−8.90	−8.70	−9.03
**4B3E**	−8.30	−8.10	−8.80	−8.40	−8.40	−8.90	−8.70	−8.60	−8.50	−8.67

**4B70**	−7.80	−8.20	−8.50	−8.00	−7.30	−7.70	−8.20	−8.00	−8.50	−8.23
**4BAQ**	−7.40	−7.50	−8.10	−7.70	−7.30	−7.70	−7.60	−7.30	−7.50	−7.33
**4BB4**	−7.60	−8.50	−8.60	−7.70	−7.30	−7.80	−7.90	−8.40	−8.30	−7.30
**4BDS**	−9.50	−9.20	−9.70	−9.60	−9.00	−8.80	−8.70	−9.00	−9.10	−9.20
**4BEX**	−6.90	−7.00	−6.60	−7.20	−6.20	−6.90	−6.50	−6.90	−7.10	−6.80
**4BF2**	−7.30	−8.40	−7.90	−7.40	−7.40	−8.00	−7.20	−8.60	−7.90	−7.10
**4BPM**	−6.50	−6.40	−6.40	−6.30	−5.90	–	−6.10	−6.40	−6.40	−6.33
**4BTI**	−8.70	−8.40	−8.40	−8.70	−8.10	−8.20	−8.20	−8.30	−8.10	−8.70
**4BVA**	−8.00	−7.90	−8.20	−7.90	−8.40	−8.80	−8.60	−8.10	−8.20	−8.40
**4C8B**	−8.00	−8.50	−8.80	−7.80	−8.60	−9.10	−9.00	−9.00	−8.90	−8.43
**4C9W**	−8.70	−8.90	−8.50	−8.70	−8.20	−8.10	−8.50	−8.90	−8.70	−8.20
**4C9X**	−9.30	−9.50	−9.80	−9.10	−8.80	−9.10	−9.20	−9.00	−8.90	−9.27
**4CFF**	−7.70	−7.60	−8.80	−9.00	−8.50	−9.00	−8.40	−7.90	−8.90	−8.20
**4CR5**	−8.10	−7.60	−7.70	−8.20	−7.70	−7.50	−7.20	−7.70	−7.40	−7.47
**4D18**	−7.70	−7.60	−7.90	−7.70	−7.50	–	−7.60	−7.40	−7.30	0.00
**4D6Z**	−8.70	−9.20	−8.70	−8.50	−8.20	−9.30	−8.10	−8.80	−9.40	−8.67
**4D75**	−8.90	−9.20	−8.60	−8.80	−8.20	−9.90	−8.20	−8.80	−9.70	−8.70
**4D76**	−8.30	−7.40	−7.30	−8.40	−7.40	−7.60	−7.50	−7.70	−7.50	−7.20
**4D89**	−8.60	−8.70	−8.80	−9.00	−8.50	−8.20	−7.90	−8.70	−8.70	−8.60
**4DCH**	−8.00	−8.40	−8.60	−8.20	−8.30	−8.20	−7.90	−8.60	−8.60	−8.07
**4DJH**	−7.70	−7.40	−9.40	−7.60	−7.30	−7.90	−7.30	−7.60	−7.60	−7.40

**4DM6**	−7.80	−7.60	−8.10	−7.70	−7.70	−8.10	−8.30	−7.50	−7.90	−7.33
**4DM8**	−7.90	−8.40	−8.30	−7.90	−8.10	−8.30	−7.90	−8.50	−8.10	−7.70
**4DMA**	−7.30	−7.60	−8.80	−7.80	−7.50	−7.30	−7.30	−7.30	−7.50	−7.80
**4DOS**	−8.90	−8.70	−9.10	−9.40	−8.40	−8.50	−8.20	−8.10	−7.90	−8.50
**4DQM**	−8.10	−6.90	−8.60	−8.30	−7.60	−7.80	−7.90	−7.80	−7.20	−8.40
**4DRJ**	−9.40	−9.30	−10.50	−9.30	−9.40	−9.70	−8.90	−9.40	−9.10	−8.87
**4DVF**	−7.40	−6.90	−7.90	−7.20	−7.20	−7.50	−6.80	−7.20	−7.20	−7.10
**4DY0**	−7.10	−7.10	−7.60	−7.30	−7.10	−7.60	−7.40	−7.40	−7.20	−6.97
**4DYM**	−7.60	−8.80	−9.20	−7.80	−7.80	−7.90	−7.50	−9.80	−9.00	−7.43
**4E2J**	−7.80	−8.00	−8.60	−7.70	−7.80	−8.80	−8.40	−8.10	−8.60	−9.00
**4EBV**	−7.50	−6.90	−7.30	−7.60	−6.90	−7.70	−7.60	−7.60	−7.80	−7.70
**4EK4**	−6.90	−6.90	−7.30	−7.30	−6.90	–	−7.10	−7.10	−7.00	−6.80
**4EKG**	−7.50	−7.50	−7.70	−7.30	−7.70	−7.60	−7.70	−7.60	−8.10	−7.70
**4EM9**	−8.00	−8.00	−8.30	−8.10	−7.80	−7.50	−7.50	−7.90	−8.50	−8.37
**4EMA**	−8.00	−7.90	−8.30	−8.20	−7.60	−8.60	−7.70	−7.70	−8.40	−7.73
**4EY4**	−8.30	−8.10	−8.00	−8.50	−8.20	−8.50	−7.50	−8.20	−8.40	−8.43
**4EY5**	−8.30	−8.60	−8.00	−8.40	−7.80	−8.50	−7.60	−8.20	−8.70	−8.43
**4EY6**	−8.10	−8.30	−7.90	−8.20	−7.80	−8.30	−7.40	−8.10	−8.40	−7.97
**4EY7**	−8.40	−8.10	−8.00	−8.50	−8.10	−8.30	−8.20	−8.40	−8.30	−8.33
**4FA2**	−8.40	−8.40	−8.70	−8.40	−7.90	−7.90	−7.60	−9.70	−8.80	−7.70
**4FM9**	−8.70	−9.10	−9.10	−9.00	−9.20	−9.30	−9.40	−8.80	−9.50	−8.97

**4FVT**	−8.90	−9.80	−8.40	−9.00	−8.20	−8.70	−8.30	−7.60	−9.10	−8.30
**4FWF**	−8.00	−7.60	−8.30	−8.00	−7.60	−7.60	−7.50	−7.80	−7.80	−7.90
**4FYR**	−8.90	−9.40	−9.40	−9.50	−8.50	−8.60	−8.40	−9.60	−9.50	−8.17
**4G5J**	−7.30	−7.40	−7.90	−7.30	−7.30	−7.20	−7.40	−7.50	−7.50	−7.27
**4GQS**	−7.70	−7.30	−8.60	−7.80	−7.50	−8.20	−7.80	−7.30	−7.40	−7.97
**4GU0**	−8.20	−8.10	−8.60	−8.70	−8.50	−8.20	−8.20	−8.60	−8.40	−8.70
**4HLW**	−7.20	−7.90	−7.90	−7.20	−7.20	−7.70	−6.80	−7.50	−8.40	−7.53
**4I0F**	−7.60	−7.60	−7.60	−7.90	−7.90	−7.70	−8.10	−7.40	−8.00	−8.20
**4I5I**	−7.70	−7.50	−7.90	−8.20	−7.50	−7.60	−7.90	−7.90	−7.90	−8.13
**4IDT**	−7.80	−8.60	−9.00	−7.40	−8.00	−9.20	−8.90	−9.20	−9.40	−8.40
**4IEH**	−7.10	−6.80	−7.70	−7.10	−7.30	−7.10	−7.10	−7.20	−7.40	−7.40
**4IP9**	−7.00	−7.30	−7.50	−7.10	−7.20	−7.00	−7.20	−7.30	−7.40	−7.40
**4IS8**	−7.90	−7.60	−8.50	−9.70	−8.70	−8.90	−8.40	−8.40	−7.50	−8.13
**4ISE**	−8.00	−8.10	−8.60	−7.90	−7.90	−8.10	−7.80	−8.70	−8.40	−7.90
**4ISF**	−8.00	−8.00	−8.10	−7.60	−8.00	−7.60	−7.70	−8.10	−8.40	−8.00
**4ISG**	−7.40	−7.50	−7.70	−7.50	−7.40	−7.50	−7.80	−8.00	−7.80	−7.70
**4IWV**	−8.30	−7.90	−8.10	−8.10	−7.90	−8.20	−8.30	−7.80	−8.30	−7.83
**4IXC**	−7.70	−7.70	−7.80	−7.60	−8.40	−8.10	−7.70	−7.70	−8.20	−7.57
**4IZY**	−8.30	−7.50	−8.00	−7.80	−7.70	−7.30	−7.10	−7.70	−7.80	−7.13
**4J1Y**	−7.20	−7.40	−7.20	−7.40	−7.40	−7.40	−7.20	−7.70	−7.00	−6.90
**4J52**	−8.60	−8.40	−8.90	−9.00	−7.70	−7.70	−7.70	−8.90	−8.30	−7.80

**4J8M**	−8.00	−7.90	−8.10	−7.90	−7.90	–	−7.40	−8.30	−7.90	−8.20
**4JNK**	−8.40	−8.20	−9.00	−8.10	−7.80	–	−7.70	−8.30	−8.80	−7.80
**4JPS**	−8.00	−8.80	−8.90	−8.70	−8.80	−8.70	−8.50	−8.80	−8.40	−8.30
**4JZF**	−7.30	−6.90	−7.20	−7.30	−6.90	−7.80	−6.80	−7.30	−7.00	−6.90
**4K9W**	−9.00	−8.40	−8.90	−9.20	−8.50	−9.00	−8.50	−8.60	−8.70	−8.50
**4KGH**	−6.50	−6.40	−6.50	−6.70	−6.40	−6.40	−6.70	−6.30	−6.80	−6.53
**4KMN**	−6.20	−6.30	−6.70	−6.40	−6.00	−5.80	−5.60	−6.30	−6.40	−6.30
**4KMP**	−7.50	−6.90	−7.80	−7.00	−6.90	−6.90	−6.70	−8.20	−7.40	−7.30
**4KXQ**	−7.60	−7.60	−7.60	−7.00	−7.40	−7.40	−7.00	−7.20	−7.90	−7.60
**4L02**	−8.40	−8.50	−8.20	−9.00	−7.70	−8.20	−8.20	−8.30	−7.70	−8.30
**4L3Q**	−8.20	−8.00	−8.20	−8.70	−8.00	−8.40	−7.60	−7.80	−8.10	−7.70
**4LGE**	−6.70	−6.80	−6.60	−6.40	−6.50	−6.60	−6.20	−7.00	−6.70	−6.43
**4LNW**	−7.90	−7.80	−7.60	−7.90	−7.70	−8.10	−7.60	−8.10	−8.30	−8.07
**4LNX**	−7.50	−8.00	−7.40	−8.00	−7.00	−7.50	−7.00	−7.30	−7.70	−7.20
**4LOG**	−7.30	−7.40	−8.00	−8.00	−7.70	−8.10	−7.20	−7.10	−7.50	−8.00
**4LVT**	−7.90	−7.60	−7.90	−7.70	−7.40	−7.60	−7.30	−8.00	−7.70	−7.80
**4LXZ**	−7.90	−7.90	−8.30	−8.00	−7.80	−8.60	−8.70	−8.10	−8.00	−7.80
**4LY1**	−7.80	−7.40	−8.20	−7.80	−8.00	−8.20	−7.60	−7.70	−8.10	−7.70
**4M8E**	−7.40	−6.90	−7.10	−7.50	−6.80	−6.90	−6.30	−7.10	−7.20	−6.90
**4M8H**	−7.70	−6.90	−7.70	−7.60	−6.80	−6.90	−6.40	−7.20	−7.10	−6.50
**4MAN**	−7.80	−7.60	−8.00	−7.70	−7.60	−7.70	−7.70	−7.50	−7.50	−8.10

**4MDN**	−7.80	−7.30	−7.30	−7.50	−6.90	−7.10	−7.10	−7.00	−7.20	−7.00
**4MG5**	−7.50	−7.10	−8.60	−8.40	−7.70	−7.50	−7.40	−7.50	−7.40	−7.50
**4MG6**	−9.20	−8.60	−8.60	−8.40	−7.60	−8.00	−7.80	−7.80	−7.80	−7.70
**4MG7**	−8.70	−7.50	−9.00	−9.10	−8.00	−7.80	−7.10	−7.60	−8.00	−8.37
**4MG8**	−7.50	−8.00	−8.60	−7.30	−7.40	−7.50	−7.70	−7.40	−7.80	−7.40
**4MG9**	−7.60	−7.60	−8.20	−7.60	−7.30	−7.50	−7.50	−7.20	−7.90	−7.80
**4MGA**	−7.60	−7.60	−8.50	−7.70	−7.40	−7.60	−7.60	−7.50	−7.90	−7.50
**4MGB**	−8.30	−7.90	−8.20	−8.30	−7.70	−8.20	−7.70	−7.90	−7.80	−7.80
**4MGC**	−7.40	−7.50	−8.60	−7.80	−7.20	−7.90	−7.40	−7.20	−8.00	−7.37
**4MGD**	−7.90	−7.90	−8.10	−8.20	−7.20	−7.80	−7.60	−7.50	−8.00	−7.87
**4MJH**	−9.80	−9.90	−10.90	−9.50	−9.50	−9.90	−9.60	−9.00	−9.00	−8.90
**4MLE**	−8.10	−8.20	−8.20	−8.00	−8.00	−8.20	−8.20	−8.40	−8.20	−7.77
**4MLH**	−7.90	−8.60	−8.00	−8.00	−8.10	−8.50	−8.00	−8.00	−8.50	−8.10
**4MXO**	−7.90	−7.90	−8.60	−8.10	−8.00	−7.80	−7.30	−8.30	−7.70	−7.60
**4N1T**	−6.20	−6.80	−6.70	−6.20	−6.70	−7.30	−6.90	−6.80	−7.00	−6.10
**4N1U**	−7.90	−7.70	−8.60	−7.90	−7.60	−8.30	−7.90	−7.00	−7.80	−8.40
**4NA7**	−8.10	−7.60	−8.00	−8.30	−7.60	−7.40	−7.50	−8.20	−7.50	−7.00
**4NA8**	−7.80	−7.10	−8.00	−8.20	−7.60	−7.30	−7.10	−7.40	−7.20	−6.87
**4NJD**	−7.50	−7.60	−7.50	−7.40	−7.00	−7.00	−6.90	−7.90	−7.60	−7.93
**4NO7**	−7.20	−7.10	−7.20	−7.40	−7.50	−7.30	−7.60	−7.20	−7.70	−7.30
**4NOS**	−8.50	−8.70	−10.40	−8.30	−7.80	−8.30	−8.00	−8.40	−9.80	−8.57

**4NRE**	−8.20	−7.80	−9.00	−8.10	−8.20	–	−8.30	−7.80	−8.50	−8.27
**4OAR**	−8.20	−8.20	−8.30	−8.30	−8.00	−8.10	−8.10	−8.40	−8.50	−8.50
**4OGH**	−7.40	−8.80	−7.60	−7.20	−7.20	−8.00	−7.20	−8.10	−9.20	−7.93
**4OM7**	−7.90	−8.30	−8.20	−8.00	−9.10	–	−8.80	−8.20	−8.50	−8.30
**4OYA**	−7.60	−8.20	−8.50	−7.50	−7.60	−9.00	−8.00	−8.30	−8.10	−8.50
**4PNZ**	−8.20	−7.70	−8.10	−7.60	−7.70	−7.80	−7.70	−7.70	−7.90	−7.90
**4PRG**	−7.60	−8.80	−8.40	−8.30	−7.80	−8.40	−7.90	−8.10	−7.90	−8.07
**4PVU**	−7.30	−7.50	−8.30	−7.50	−6.90	−7.70	−7.70	−7.90	−7.90	−7.37
**4PWL**	−8.30	−7.60	−8.80	−7.80	−7.60	−7.70	−7.30	−7.50	−7.70	−7.87
**4PZW**	−8.30	−8.90	−9.50	−8.40	−7.80	−8.40	−8.40	−9.00	−9.20	−8.50
**4Q9S**	−6.70	−7.90	−7.90	−8.00	−6.80	−7.10	−6.90	−7.90	−8.20	−6.77
**4QBZ**	−8.80	−8.40	−8.70	−8.60	−8.60	–	−8.10	−8.60	−9.10	−8.40
**4QC0**	−8.60	−8.60	−8.90	−8.70	−8.50	–	−8.20	−9.20	−8.70	−8.67
**4QTD**	−7.90	−8.60	−8.50	−8.40	−8.20	–	−8.50	−9.70	−8.90	−8.00
**4QVX**	−6.90	−7.70	−7.30	−6.80	−6.60	−7.50	−6.30	−8.70	−8.10	−7.70
**4R07**	−8.40	−8.40	−8.60	−8.50	−8.00	–	−8.60	−8.70	−9.00	−7.93
**4R08**	−8.60	−8.50	−8.30	−8.40	−8.50	–	−8.70	−8.80	−9.00	−8.50
**4R09**	−8.50	−8.30	−8.30	−8.20	−8.00	–	−8.20	−9.10	−8.60	−8.27
**4R0A**	−8.30	−8.00	−8.70	−8.80	−8.00	–	−7.40	−8.50	−8.80	−7.87
**4R3C**	−7.70	−7.60	−7.90	−7.70	−7.80	–	−7.70	−8.20	−7.90	−7.70
**4R6A**	−9.00	−8.90	−8.70	−8.90	−8.70	–	−8.50	−9.20	−8.90	−9.00

**4R7H**	−6.80	−7.20	−7.00	−6.70	−7.10	−7.60	−7.30	−7.30	−7.90	−7.73
**4RCH**	−8.10	−8.10	−8.70	−8.10	−8.20	−8.40	−7.90	−7.80	−8.40	−7.70
**4RG2**	−7.10	−7.60	−7.70	−7.30	−7.10	–	−6.80	−7.70	−7.20	−7.57
**4THN**	−8.50	−7.40	−8.00	−8.70	−7.20	–	−7.40	−6.60	−6.90	−7.47
**4TPK**	−9.20	−9.40	−9.70	−9.20	−9.20	−8.80	−9.40	−8.80	−9.10	−9.03
**4TUH**	−8.20	−8.20	−8.30	−8.40	−8.00	−8.40	−8.50	−9.20	−8.40	−8.13
**4TUZ**	−7.60	−7.00	−8.50	−8.00	−7.50	−7.60	−8.00	−7.60	−7.80	−7.53
**4TV1**	−7.60	−7.10	−8.20	−8.10	−7.30	−7.80	−7.60	−7.40	−7.40	−7.47
**4TW8**	−8.10	−7.40	−8.10	−7.80	−7.60	−8.60	−7.50	−7.50	−7.50	−7.57
**4U7T**	−7.30	−7.50	−7.70	−7.90	−7.50	−7.40	−7.10	−7.70	−7.50	−7.63
**4UDB**	−8.50	−7.50	−8.80	−7.70	−7.70	−7.60	−8.00	−7.00	−7.90	−8.27
**4UDC**	−7.70	−7.70	−8.30	−7.60	−7.30	−7.70	−7.30	−6.90	−7.80	−7.67
**4UDD**	−7.70	−7.60	−8.20	−7.70	−7.40	−7.90	−7.90	−7.10	−7.80	−8.20
**4UE7**	−7.20	−7.50	−7.60	−7.80	−6.80	−7.10	−7.60	−7.00	−7.10	−7.60
**4URV**	−8.10	−7.10	−7.70	−7.70	−7.50	−7.70	−7.80	−7.40	−7.30	−7.17
**4W93**	−8.40	−8.80	−8.50	−8.80	−8.40	−8.90	−8.90	−8.20	−8.80	−8.80
**4W9P**	−8.60	−8.40	−9.10	−8.60	−7.30	−8.30	−7.80	−9.30	−8.60	−7.93
**4WH9**	−7.20	−6.90	−7.70	−7.40	−7.40	−7.40	−7.10	−7.90	−7.00	−6.90
**4WT2**	−9.00	−7.70	−8.30	−8.90	−7.80	−7.90	−7.50	−7.70	−9.00	−8.00
**4WUA**	−7.50	−8.40	−8.40	−8.10	−7.60	−7.80	−7.40	−8.30	−7.90	−7.47
**4WVS**	−6.20	−6.40	−6.40	−6.20	−5.60	−6.10	−6.20	−6.70	−6.20	−5.67

**4WVT**	−7.70	−7.20	−8.20	−8.00	−7.20	−7.40	−7.20	−7.80	−8.60	−7.13
**4WZV**	−7.80	−7.50	−7.60	−7.40	−7.30	−7.90	−7.80	−7.80	−7.70	−8.07
**4X30**	−7.40	−6.80	−6.80	−7.50	−6.60	−7.20	−6.90	−7.20	−7.00	−7.20
**4X9Y**	−8.30	−8.50	−8.20	−8.40	−7.60	−8.20	−8.00	−8.00	−8.20	−7.50
**4XCT**	−7.00	−7.10	−7.70	−7.00	−7.50	−7.40	−7.40	−7.50	−7.60	−8.03
**4XII**	−9.40	−9.60	−10.20	−9.90	−9.60	−9.20	−10.00	−9.30	−9.20	−9.43
**4XRY**	−7.70	−8.20	−9.60	−8.40	−8.10	−8.70	−8.10	−7.70	−8.80	−8.40
**4Y72**	−8.20	−7.70	−7.60	−8.50	−7.30	−7.50	−7.50	−7.60	−7.40	−8.20
**4ZH8**	−8.40	−8.00	−7.70	−8.20	−7.50	−7.30	−7.40	−7.60	−7.30	−8.40
**4ZSA**	−7.20	−7.30	−8.00	−7.40	−7.20	–	−7.00	−7.20	−7.80	−7.77
**4ZSH**	−7.70	−7.00	−7.20	−7.10	−7.00	−6.90	−6.60	−7.10	−6.90	−7.10
**4ZZN**	−7.60	−7.80	−7.80	−7.80	−7.50	–	−7.20	−7.90	−7.70	−7.40
**5ANS**	−8.10	−9.30	−9.30	−8.80	−8.60	−8.70	−8.90	−9.50	−9.60	−8.60
**5ANT**	−8.20	−7.40	−9.70	−7.60	−7.40	−9.10	−8.70	−7.70	−7.70	−8.63
**5ANU**	−6.50	−6.90	−7.40	−6.50	−6.10	−7.30	−6.70	−6.70	−7.20	−6.40
**5ANV**	−7.20	−8.90	−9.30	−6.90	−7.00	−8.40	−8.50	−7.70	−8.40	−8.30
**5ANW**	−7.60	−7.10	−7.80	−6.70	−6.50	−6.80	−6.60	−6.80	−7.80	−7.27
**5AWB**	−7.90	−8.40	−7.90	−7.80	−7.70	–	−7.10	−7.70	−8.90	−7.60
**5AWD**	−8.10	−8.10	−8.40	−8.40	−7.90	–	−7.30	−7.80	−8.80	−7.60
**5AWK**	−7.80	−8.30	−8.30	−7.70	−7.60	−8.50	−8.40	−8.30	−7.30	−8.40
**5B75**	–	−8.80	−8.90	–	–	–	–	–	–	–

**5C3H**	−6.20	−6.50	−6.70	−6.30	−5.90	−6.40	−5.80	−6.40	−6.50	−6.20
**5C91**	−7.40	−7.80	−7.80	−7.50	−7.70	–	−7.50	−7.40	−7.90	−7.80
**5CJ6**	−7.30	−7.10	−7.50	−7.40	−7.30	−7.60	−7.40	−7.20	−7.70	−7.27
**5CR1**	−7.10	−7.30	−7.30	−7.00	−7.00	−8.20	−7.30	−7.20	−6.90	−7.10
**5CSW**	−7.40	−8.70	−8.20	−7.60	−7.40	–	−7.50	−8.50	−8.70	−8.00
**5DCP**	−7.30	−7.00	−7.80	−7.30	−6.70	−7.10	−7.00	−7.50	−6.90	−7.10
**5E6H**	−7.50	−9.30	−8.40	−7.70	−8.30	−8.40	−8.20	−7.70	−8.90	−7.67
**5EGM**	−7.80	−8.60	−8.60	−8.00	−7.20	−7.30	−7.20	−8.60	−8.10	−8.10
**5EXO**	−6.70	−6.70	−6.30	−6.80	−6.30	−6.70	−6.10	−6.70	−6.50	−6.40
**5FC4**	−6.90	−6.20	−7.00	−6.70	−6.40	−6.40	−6.50	−6.20	−6.90	−5.83
**5FNC**	−9.00	−9.00	−9.20	−9.10	−8.00	–	−7.90	−7.60	−9.60	−7.90
**5FQT**	−7.40	−7.40	−9.20	−7.20	−7.30	−6.80	−6.60	−6.70	−7.00	−7.17
**5G3J**	−7.30	−7.30	−9.00	−7.10	−7.20	−7.80	−8.30	−6.60	−7.80	−7.93
**5G5W**	−7.40	−7.50	−7.10	−7.40	−6.70	−7.30	−7.70	−7.20	−7.90	−7.57
**5GPG**	−9.30	−10.00	−9.90	−9.40	−9.10	−9.00	−9.10	−9.10	−9.40	−9.00
**5GT4**	−7.20	−9.00	−9.20	−7.40	−7.40	−8.90	−9.60	−7.50	−8.50	−8.87
**5H1D**	−6.90	−7.80	−7.00	−7.20	−6.70	−7.00	−6.70	−6.80	−6.90	−7.00
**5HDH**	−8.30	−7.70	−9.70	−8.70	−7.70	–	−7.40	−9.50	−8.40	−7.70
**5HG8**	−7.60	−7.30	−8.30	−7.50	−7.60	–	−7.00	−7.80	−7.50	−7.90
**5HK1**	−6.80	−6.90	−7.60	−7.40	−7.00	−7.80	−7.40	−7.00	−7.10	−7.57
**5I7P**	−7.30	−6.80	−7.00	−7.20	−6.10	−6.30	−6.00	−6.60	−6.70	−6.60

**5IEZ**	−7.50	−7.10	−8.70	−7.40	−7.20	−7.70	−7.30	−7.30	−7.80	−7.27
**5ITU**	−8.00	−8.10	−8.60	−9.30	−7.90	−8.30	−7.90	−8.40	−8.20	−7.87
**5IXS**	−8.40	−7.40	−8.80	−7.90	−8.10	–	−8.20	−8.10	−8.50	−7.77
**5J20**	−8.50	−8.00	−8.80	−7.90	−7.40	−7.80	−6.80	−7.80	−7.70	−7.10
**5J2X**	−6.50	−6.40	−6.60	−6.70	−6.40	−6.90	−6.30	−6.40	−6.50	−6.40
**5J6N**	−6.80	−7.10	−7.30	−7.20	−7.40	−7.60	−7.50	−7.20	−7.10	−6.77
**5JFR**	−7.70	−7.80	−8.70	−8.30	−7.60	−8.20	−7.10	−8.30	−7.40	−7.17
**5K5E**	−9.10	−9.30	−9.80	−9.70	−9.10	−8.80	−8.90	−9.00	−9.40	−9.17
**5KBY**	−8.30	−7.70	−8.00	−8.10	−7.40	−7.70	−7.60	−7.70	−7.70	−7.80
**5KDI**	−8.30	−8.00	−8.60	−8.50	−7.70	−8.20	−7.60	−7.90	−7.90	−7.83
**5KXA**	−7.50	−7.80	−8.50	−8.30	−7.80	−8.10	−7.80	−7.90	−8.60	−8.03
**5KYJ**	−8.20	−8.00	−8.30	−8.00	−7.80	−7.90	−8.30	−7.80	−8.00	−7.77
**5L9B**	−7.40	−7.50	−7.40	−7.30	−6.50	−7.40	−6.80	−6.60	−7.20	−7.10
**5LOF**	−8.50	−8.00	−9.10	−8.60	−8.00	−9.40	−9.30	−8.60	−9.20	−8.50
**5NJK**	−7.70	−7.60	−8.00	−7.40	−7.40	−7.50	−7.90	−7.60	−7.60	−7.73
**5NJX**	−6.70	−6.90	−7.30	–	–	−7.00	–	–	–	−7.00
**5NZO**	−7.40	−6.80	−7.30	−7.20	−6.50	–	−6.20	−7.30	−7.20	−7.33
**5NZP**	−7.60	−6.80	−7.30	−7.20	−6.70	–	−6.80	−7.10	−7.10	−7.20
**5OMP**	−7.60	−6.80	−7.90	–	–	−7.20	–	–	–	−6.97
**5ORL**	−7.60	−8.00	−8.80	−7.60	−6.70	−8.60	−7.30	−9.20	−9.10	−7.37
**5T8E**	−7.40	−7.30	−7.20	−7.30	−7.60	−7.60	−6.70	−7.90	−7.70	−7.43

**5TKS**	−8.30	−7.40	−7.60	−8.40	−7.50	−7.20	−7.10	−8.10	−7.30	−7.10
**5TQ3**	−8.30	−8.40	−8.70	−8.20	−8.50	−8.30	−8.10	−7.90	−8.20	−8.13
**5TQE**	−7.70	−6.80	−7.20	−7.50	−7.00	−7.10	−6.60	−6.80	−6.90	−6.53
**5TWZ**	−7.20	−7.10	−8.10	−7.70	−8.10	−8.00	−7.60	−7.70	−7.40	−7.43
**5UAK**	−7.50	−6.80	−7.90	−7.20	−7.30	−7.90	−7.30	−7.60	−7.30	−7.70
**5UCH**	−7.70	−7.60	−8.00	−7.80	−7.40	−7.50	−7.50	−8.20	−7.80	−8.00
**5UF9**	−7.20	−7.30	−8.30	−8.20	−7.60	−7.10	−7.20	−7.60	−7.70	−7.97
**5UG8**	−7.00	−7.70	−7.60	−7.70	−7.30	–	−7.40	−7.70	−7.80	−7.47
**5VAD**	−7.90	−7.50	−8.30	−7.80	−8.20	–	−8.30	−8.10	−7.80	−7.70
**6ASY**	−7.70	−7.60	−8.20	−7.90	−7.30	−7.70	−7.40	−8.20	−7.70	−7.63
**6BCA**	−7.00	−7.30	−7.90	−7.30	−6.80	−6.90	−6.60	−6.70	−7.30	−7.20

Table S2. Additional information for the 10 proteins with highest affinity score Table 2.

Gene	Uniprot ID	Protein	Class
NTRK1	P04629	High affinity nerve growth factor receptor	2
HSPB1	P04792	Heat shock protein beta−1	1
NOS3	P29474	Nitric oxide synthase, endothelial	4
MTOR	P42345	Serine/threonine−protein kinase mTOR	2
NOS2	P35228	Nitric oxide synthase, inducible	4
RORA	P35398	Nuclear receptor ROR−alpha	3
AGER	Q15109	Glycosylation end product−specific receptor	5
BCHE	P06276	Cholinesterase	6
DHFR	P00374	Dihydrofolate reductase	4
AKR1C3	P42330	Aldo−keto reductase family 1 member C3	4

Class: 1. Chaperone; 2. Isomerase/Transferase; 3. Lipid binding protein; 4. Oxido reductase; 5. Transport protein/Signaling protein; 6. Hidrolase

Table S3. Node code and respective name for protein in Figure 3.

NODE CODE	PROTEIN NAME
HSP90AB1	Heat shock protein HSP 90−beta
DHFR	Dihydrofolate reductase
NUDT1	7,8−dihydro−8−oxoguanine triphosphatase
SERPINE1	Plasminogen activator inhibitor 1
ANPEP	Aminopeptidase N
FKBP4	Peptidyl−prolyl cis−trans isomerase FKBP4
BCHE	Cholinesterase
MCL1	Induced myeloid leukemia cell differentiation protein Mcl−1
RXRA	Retinoic acid receptor RXR−alpha; Receptor for retinoic acid.
HSD17B1	Estradiol 17−beta−dehydrogenase 1
NOS2	Nitric oxide synthase, inducible
RXRB	Retinoic acid receptor RXR−beta
FKBP3	Peptidyl−prolyl cis−trans isomerase FKBP3
NOS3	Nitric oxide synthase, endothelial
CLK1	Dual specificity protein kinase CLK1
AKR1C3	Aldo−keto reductase family 1 member C3
PCSK9	Proprotein convertase subtilisin/kexin type 9
TOP2A	DNA topoisomerase 2−alpha
CYP3A4	Cytochrome P450 3A4
RORC	Nuclear receptor ROR−gamma
RORA	Nuclear receptor ROR−alpha
AGER	Advanced glycosylation end product−specific receptor
NR5A2	Nuclear receptor subfamily 5 group A member 2
HSPB1	Heat shock protein beta−1
NTRK1	High affinity nerve growth factor receptor
ACVR1	Activin receptor type−1
HSP90AA1	Heat shock protein HSP 90−alpha
HSD11B1	Corticosteroid 11−beta−dehydrogenase isozyme 1
TXNRD1	Thioredoxin reductase 1, cytoplasmic
AKT2	RAC−beta serine/threonine−protein kinase
NR1H2	Oxysterols receptor LXR−beta
AKT1	RAC−alpha serine/threonine−protein kinase

3. PPI NETWORK ANALYSIS

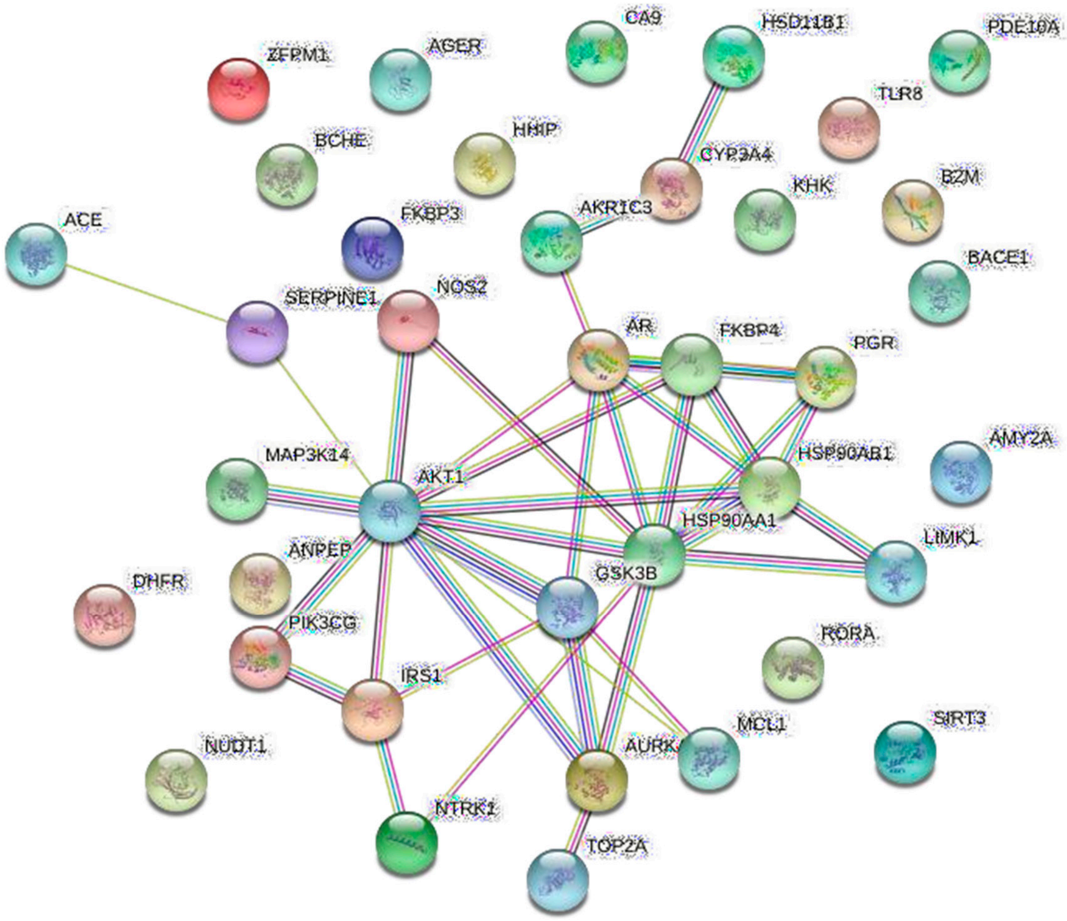

Figure S1. Predicted protein interaction network for complexes with voacristine alkaloid (PPI enrichment *p*-value: 1.47 e10−13/Minimum required interaction score: High confidence –0.700). Edges represent the evidence of protein-protein associations. Nodes represent proteins with good binding affinity energies (lower than −9.0 kcal/mol) with the molecule.

The results obtained for the best protein complexes with the alkaloid voacristine showed weak interactions with each other. Just 2 nodes are involved in physiological processes of alkaloid metabolism, however, the signaling cascades are independent. The HSP90AA1 are positioned as the center of the PPI network, having as main action the dimerization between their Alpha and Beta units and the protection of other macromolecules such as dihydrofolate reductase (DHFR), receptor for nerve growth factor (NTRK1) and the inhibition of the ubiquitination of nitric Oxide Synthase (NOS2) (Clapp, *et al*., 2012).

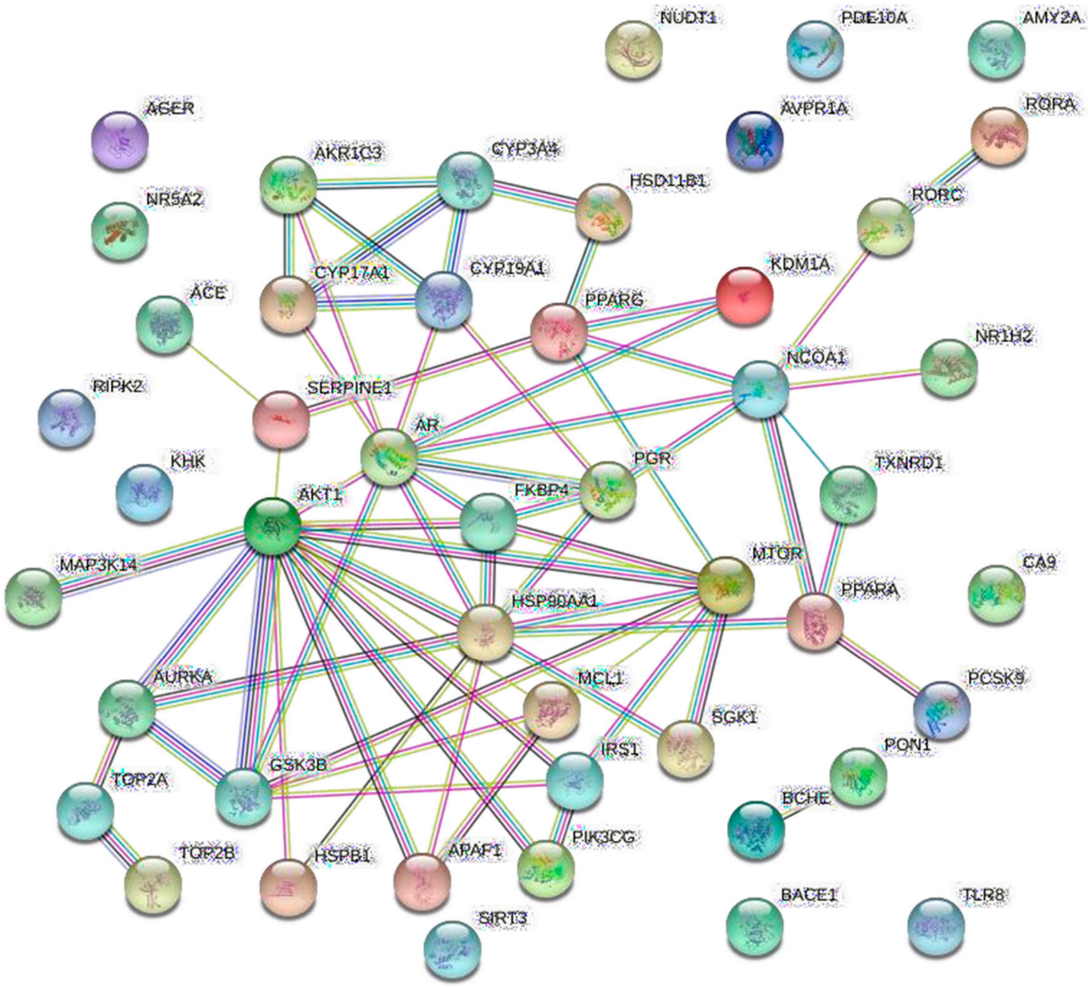

Figure S2. Predicted protein interaction network for complexes with voacangine-7-hydroxyndolenine alkaloid (PPI enrichment *p*-value: 1.0 e−16 / Minimum required interaction score: High confidence –0.700). Edges represent the evidence of protein−protein associations. Nodes represent proteins with good binding affinity energies (lower than −9.0 kcal/mol) with the molecule.

The main node of the network is found in the RAC−alpha serine/threonine−protein kinase (AKT1) protein, which has a close relationship with glycogen synthase kinase−3 beta (GSK3) and serine/threonine−protein kinase mTOR (MTOR) in signaling pathways that lead to the regulation of processes such as protein synthesis and cell cycle progression (Hermida, et al., 2017). Additionally, AKT1 regulates the expression of genes associated with cell differentiation, hormonal response and cell survival through its catalytic activity on the retinoic acid receptor RXR−beta (RXRB) and its subsequent binding as a heterodimer with oxysterols receptor LXR alpha and beta (Krężel, et al., 2019). The alkaloid voacangina-7-hidroxyndolenina has a hydroxyl group (OH) in position 7 on the indole group. Through the protein-protein

interaction network, it was shown how the presence of this group produces a response on proteins of metabolism of oxidizing agents, such as CYP3A4 monooxygenases, which we find as the second nucleus of the potential target network of voacangine-7-hydroxyndolenine. An interaction between protection proteins to the nervous system, such as serumparaoxonase/arylesterase 1 (PON1) and butirylcholinesterase (BCHE), with CYP3A4 was found (Palikov, et al., 2020). Finally, we were able to visualize other proteins with REDOX functions such as aldo−keto reductase family 1 member C3 (AKR1C3), which main function is the conversion of ketones and aldehydes to alcohols leading to the detoxification of xenobiotics.

4. Spectroscopic data for isolated alkaloids

The compound voacangine exhibited the following physical and spectral properties: Crystalline needles; Mp: 137−138 °C; R*f*: 0.8 in hexane: ethyl acetate (6:4); ^{1}H NMR (300 MHz, CDCl3): δ 7.73 (s, NH), 7.17 (d, *J*= 8.7 Hz, H−12), 6.96 (d, *J*= 2.6 Hz, H−9), 6.84 (dd, *J*= 8.6 y 2.6 Hz, H−11), 3.89 (s, OCH_3), 3.73 (s, CO_2CH_3), 0.92 (t, *J*= 7 Hz, H−18). ^{13}C NMR (75 MHz, $CDCl_3$): δ 176.03 (CO_2Me), 154.11 (C− 10),137.65 (C−2), 130.63 (C−13), 129.31 (C−8), 111.96 (C−11), 111.23 (C−12), 110.24 (C−9), 100.84 (C−7), 57.69 (C−21), 56.15 (OCH_3), 55.25 (C−16), 53.24 (C−5), 52.74 (CO_2CH_3), 51.60 (C−3), 39.27 (C−20), 36.67 (C−17), 32.14 (C−15), 27.44 (C−14), 26.86 (C−19), 22.33 (C−6), 11.82 (C−18).

The compound voacangine−7−hydroxyindolenine exhibited the following physical and spectral properties: Amorphous yellow−green solid; Mp: 135−137 °C; R*f*: 0.5 in hexane: ethyl acetate (6:4); ^{1}H NMR (300 MHz, $CDCl_3$): δ 7.36 (d, *J*= 9 Hz, H−12), 6.91 (s, H−9), 6.80 (dd, *J*= 6 y 3 Hz, H−11), 3.82 (s, OCH3), 3.70 (s, CO2CH3), 0.86 (t,*J*= 7 Hz, H−18) ppm. ^{13}C NMR (75 MHz, $CDCl_3$): δ 186.97 (C−2), 174.05 (CO_2Me), 159.23 (C−10), 144.90 (C−13), 144.53 (C−8), 121.49 (C−12), 113.85 (C−11), 108.09 (C−9), 88.43 (C−7), 58.68 (C−21), 55.88 ($ArOCH_3$; C16), 53.39 (CO_2CH_3), 49.22 (C−5), 48.75 (C−3), 37.68 (C−20), 34.63 (C−17), 34.25 (C−6), 32.14 (C−15), 27.08 (C−14), 26.62 (C−19),
11.71 (C−18).

The compound rupicoline exhibited the following physical and spectral properties: yellow crystals; Mp: 252−253°C; R*f*: 0.0 in hexane:ethyl acetate (6:4); ^{1}H NMR (300 MHz, CDCl3): δ 7.07 (dd, *J*= 8 y 3 Hz, H−11), 7.02 (s, H−9), 6.76 (d, *J*= 9 Hz, H−12), 4.29 (s, N−H), 3.95 (s, H−4), 3.76 (s, OCH_3), 3.30 (s, CO_2CH_3), 1.91 (s, H− 14), 0.91 (t, *J*= 6 Hz, H−18). ^{13}C NMR (75 MHz, $CDCl_3$): δ 202.9 (C−7), 174.57 (CO_2Me), 154.21 (C−10), 153.82 (C−13), 126.89 (C−11), 121.78 (C−8), 114.12 (C−12),

104.63 (C−9), 55.90 ($ArOCH_3$), 52.07 (C−3), 52.07 (C−16), 52.05 (C−21), 51.31
(CO_2CH_3), 47.67 (C−5), 35.81 (C−20), 31.1 (C−15), 30.8 (C−17), 28.69 (C−19), 26.08 (C−
14), 25.77 (C−6), 12.14 (C−18).

REFERENCES

1. Clapp, K. M., Peng, H. M., Jenkins, G. J., Ford, M. J., Morishima, Y., Lau, M., & Osawa, Y. (2012). Ubiquitination of neuronal nitric−oxide synthase in the calmodulin−binding site triggers proteasomal degradation of the protein.Journal of Biological Chemistry,287(51), 42601−42610.
2. Hermida, M. A., Kumar, J. D., & Leslie, N. R. (2017). GSK3 and its interactions with the PI3K/AKT/mTOR signalling network.Advances in biological regulation,65, 5−15.

3. Krężel, W., Rühl, R., & de Lera, A. R. (2019). Alternative retinoid X receptor (RXR) ligands.Molecular and cellular endocrinology,491, 110436.
4. Palikov, V. A., Palikova, Y. A., & Dyachenko, I. A. (2020). Study of protective properties of butyrylcholinesterase in acute anticholinesterase poisoning on BChE−KO and BALB/c mice.Research Results in Pharmacology,6, 41.
